# Shifting Trends in the Epidemiology and Management of Idiopathic Pulmonary Fibrosis in the Era of Evidence-Based Guidelines: a Nationwide Population Study

**DOI:** 10.1007/s44197-025-00377-y

**Published:** 2025-03-17

**Authors:** Tang-Hsiu Huang, Shen-Huan Wei, Chin-Wei Kuo, Hsin-Yu Hou, Chao-Liang Wu, Sheng-Hsiang Lin

**Affiliations:** 1 Institute of Clinical Medicine, College of Medicine, National Cheng Kung University, Tainan, Taiwan; 2 Division of Chest Medicine, Department of Internal Medicine, National Cheng Kung University Hospital, College of Medicine, National Cheng Kung University, Tainan, Taiwan; 3 Department of Biochemistry and Molecular Biology, College of Medicine, National Cheng Kung University, Tainan, Taiwan; 4 Ditmanson Medical Foundation Chia-Yi Christian Hospital, Chiayi, Taiwan; 5 Department of Public Health, College of Medicine, National Cheng Kung University, Tainan, Taiwan; 6 Biostatistics Consulting Center, National Cheng Kung University Hospital, College of Medicine, National Cheng Kung University, Tainan, Taiwan

**Keywords:** Antifibrotic agents, Immunosuppressants, Incidence, Mechanical ventilation, Mortality, Prevalence

## Abstract

**Background:**

Advances in the understanding of idiopathic pulmonary fibrosis (IPF) and international cooperation have led to the publication and subsequent updates of international practice guidelines. The impact of these guidelines, especially significant updates occurring after 2011, on IPF epidemiology and clinical practices remains relatively unexplored.

**Methods:**

This retrospective nationwide population-based study utilized the Whole-Population Datafiles (WPD) of Taiwan’s National Health Insurance Research Database that contained basic demographics, complete claim data, and causes of death for all insured persons. We refined the code-based definition to identify IPF cases from the WPD between 2011 and 2019. Independent validation confirmed the high accuracy of this definition. We analyzed the annual standardized rates of IPF incidence, prevalence, overall and IPF-specific all-cause mortality. Additionally, we examined trends in the prescription of selected medications and the proportions of patients with respiratory failure receiving invasive (IMV) and non-invasive (NIV) mechanical ventilation.

**Results:**

We included 4359 incident cases of IPF. From 2011 to 2019, the annual standardized incidence rates increased from 1.66 (95% confidence interval [CI], 1.36–1.97) to 11.35 (95% CI, 10.65–12.04) per 100,000 standard population, and the annual standardized prevalence rates increased from 1.98 (95% CI, 1.65–2.31) to 27.25 (95% CI, 26.17–28.33) per 100,000 standard population. The standardized IPF-specific all-cause mortality and respiratory failure rates remained stable. Male and older patients received IPF diagnoses more frequently, and experienced higher mortality rates, compared to their female and younger counterparts. Most deaths were attributed to respiratory causes, without significant seasonal variation. Changing trends in the management of IPF mirrored with the evolving guideline recommendations, and showed diminishing roles of immunosuppressants, growing usage of antifibrotics, and NIV usage surpassing IMV.

**Conclusions:**

Our findings reflected the longitudinal impact of the recently evolving guideline recommendations on IPF epidemiology and real-world management.

**Supplementary Information:**

The online version contains supplementary material available at 10.1007/s44197-025-00377-y.

## Introduction

Idiopathic pulmonary fibrosis (IPF) is a chronic interstitial lung disease (ILD) with a very poor prognosis [1]. IPF causes progressive fibrotic destruction of the lungs, eventually leading to respiratory insufficiency, impaired quality of life, and reduced survival, with reported medial survival ranging from 3 to 5 years after diagnosis [1–7]. In the past two decades, much progress has been made in understanding the pathogenesis of IPF and in developing treatments. Collaboration among international pulmonological societies has resulted in a series of consensus documents and evidence-based guidelines regarding IPF. These guidelines were initially published in the early 2000s and have since undergone multiple updates and revisions, particularly after 2011 [2–7]. Taiwan also established a local practice guideline [8] for IPF in Chinese, which was first published in 2015 and subsequently revised in 2020 and 2023. These guidelines have incorporated the latest research findings, standardized the nomenclature and diagnostic criteria, and provided recommendations for medical practices for IPF. Previous studies have assessed clinicians’ adherence to IPF guidelines [9, 10]. However, the impact of these guidelines on the epidemiology and management of IPF at a nationwide scale, especially following the significant revisions made since the mid-2010s, has not been well investigated [11–13].

Recent epidemiological studies from various countries and regions have frequently shown an upward trajectory in the occurrence and mortality rates of IPF [1, 14–26]. However, it has been challenging to ascertain the true public health burden of IPF, as estimates on the incidence, prevalence, and mortality rates of IPF vary greatly across different studies and regions, owing to heterogeneity in methodologies and patient databases [1, 12] Moreover, data specifically from East and Southeast Asia remain limited [18–25]. Two studies from Taiwan concordantly revealed an increase in IPF incidence and prevalence rates from 1997 through 2011 [18, 19]. Despite providing early insights into Taiwan’s IPF epidemiology, these studies had certain limitations. Since there is no national registry for IPF in Taiwan, both studies used Taiwan’s National Health Insurance (NHI) Research Database, but only smaller subsets instead of the complete claim database. They relied mainly on diagnostic codes, either with (narrow definition) or without (broad definition) relevant NHI procedural claims, to identify cases of IPF. This approach was not validated by the authors and risked potential misclassification. Moreover, IPF typically affects older adults [1–3, 6, 7], but these studies set a low inclusion age limit (≥ 18 years), potentially increasing the likelihood of including non-IPF cases. Notably, both studies focused primarily on epidemiological findings and did not explore temporal changes in diagnostic and therapeutic practices for IPF. Furthermore, their timeframes preceded the publication of Taiwan’s local IPF guidelines and major revisions to international guidelines. As a result, the potential longitudinal impact of these guidelines remains unclear.

In this nationwide study, we aimed to address these unresolved questions related to the epidemiological and therapeutic impact of IPF practice guidelines. We overcame the limitations of previous studies by involving a study period that was contemporaneous with the recent evolution of the guidelines, by applying a set of independently-validated inclusion criteria [12], and by utilizing the Whole-Population Datafiles (WPD) from Taiwan’s NHI Research Database.

## Methods

### Study Design, Population, and Data Source

This retrospective nationwide population-based study utilized the WPD of Taiwan’s NHI Research Database. The study received approval from the Institutional Review Board of our hospital (approval codes A-ER-109-321 and B-EX-111-034). Authorized access to the WPD was granted by Taiwan’s Health and Welfare Data Science Center, Ministry of Health and Welfare. During the study period from 2011 through 2019, the NHI provided coverage for 99.51% to 99.84% of Taiwan’s entire population, which exceeded 23 million individuals [27]. The WPD contained anonymized data related to sex, age, and the comprehensive records of claims for individual insured persons. Specifically, relevant data were retrieved from the following WPD subfiles: Health-01 and Health-02 containing information on the sites, dates (including the dates of hospital admission and discharge), main attributing diagnostic codes, major operative procedures, and outcomes of inpatient, ambulatory, and emergent encounters; Health-04 and Health-05 containing details of inpatient, ambulatory, and emergent medical orders of intervention (such as the timing and frequencies of specific diagnostic and therapeutic procedures, modes and duration of mechanical ventilation) and medicinal prescriptions (route, dosage, and duration of individual drugs used; branded products in different dosing forms of the same generic drug are represented by distinct medication codes); Health-07 containing administrative registry data such as sex, birth years and months, city of residence, status of health insurance for all insured persons; and Health-10 containing dates, places, and causative diagnosis of death as specified on death certificates. For every insured person, data from different subfiles were crosslinked and integrated using a uniquely encrypted identity code.

### Working Definition of IPF

To be considered to have IPF, an insured person had to meet all the following criteria: (1) at least one inpatient or ambulatory (including emergent) claim with compatible International Classification of Diseases, Ninth and Tenth Revision, Clinical Modification (ICD-9-CM and ICD-10-CM) codes for IPF (specifically, the code was 516.3 or 516.31 in ICD-9-CM, or after January 1, 2016, J84.112 in ICD-10-CM) [18, 19, 28] between 2011 and 2019; (2) age 50 years or older when the diagnostic code claim was first made (as pulmonary fibrosis occurring at younger ages is likely to be non-idiopathic and secondary to a triggering etiology) [1–3, 6, 29]; (3) within one year before the initial diagnostic code claim, there had to be at least one claim for a crucial diagnostic procedure [18, 19, 28] (as listed in Supplemental Table 1); (4) no competing diagnostic code (which would change the diagnosis to a non-IPF interstitial pneumonitis, as specified in Supplemental Table 2) could be claimed within one year after the initial diagnostic code claim [2, 3, 6, 7, 30].

### Validation of the IPF Definition

We conducted an independent validation of the definition for IPF for this study using electronic medical records of our hospital. Details and a flowchart of the validation process are presented in Appendix 1 of the Supplemental Material. Briefly, from all patients who visited our hospital between 2010 and 2020, we identified 376 patients with at least one inpatient or ambulatory diagnosis of IPF based on compatible ICD-9/-10-CM codes. A panel consisting of two pulmonologists, one radiologist, and one rheumatologist meticulously reviewed the medical records, laboratory and spirometric data, and radiographic images of each screened patient. Ultimately, the panel considered 182 out of the 376 screened patients as having true IPF. When applying the aforementioned working definition established for this study, we excluded 198 patients and identified the remaining 178 patients as representing “IPF cases”. This classification resulted in 168, 184, 10, and 14 true-positive, true-negative, false-positive, and false-negative cases, respectively. Therefore, the sensitivity, specificity, positive predictive value, and negative predictive value of the IPF definition for this study were 92%, 95%, 94%, and 93%, respectively.

### Data Collection

For all included patients, we retrieved from the WPD data related to the prescription of the following medications (administered orally or intravenously) during the study period using specific NHI medication codes: corticosteroids, azathioprine, cyclophosphamide, cyclosporin, nintedanib, and pirfenidone. We standardized the dosage of different forms of corticosteroids using the following formula [31]:

(1 × E) = (A/6.7) = (B/5.3) = (C/33.3) = (D/26.7). Here, the capital letters A, B, C, D, E denote the dosages (in milligrams) of prednisolone/prednisone, methylprednisolone, cortisone, hydrocortisone, and dexamethasone, respectively. Furthermore, we retrieved data related to the use of invasive and non-invasive mechanical ventilation, using specific NHI procedural codes (Supplemental Table 1). For patients who died during the study period, we obtained information on the causes of death based on specific ICD-9/-10-CM codes in the WPD. These causes were then classified into 13 etiologic categories, including non-neoplastic respiratory conditions, other non-respiratory infections, lung cancer, other extra-pulmonary cancers, hematologic disorders, cardiovascular diseases, neurologic conditions, metabolic disorders, gastrointestinal issues, hepatobiliary diseases, nephrological-genitourinary conditions, musculoskeletal disorders, and cases relate to suicide or trauma.

### Statistical Analysis

We defined an incident case of a study year as a case that newly met the aforementioned IPF definition in that specific year. To establish a baseline, we designated the year 2010 as the “washout year”, excluding patients who had already met the definition in 2010 or earlier from the calculation of incidence rates. However, these patients would still be included in the calculation of other epidemiological quantities if they survived into the study period. Patients whose initial diagnostic code claim for IPF occurred in 2019 were followed until the end of 2020 to ensure compliance with the aforementioned criteria 4. Prevalent cases for a given study year were defined as the total number of cases meeting the IPF definition and still alive by July 1 of that same year. Patients who met the IPF definition but died from any cause during a study year were considered as the all-cause mortal cases of that year. We performed direct standardization to calculate the standardized incidence, prevalence, and all-cause mortality rates using the sex and 10-year-wide age-subgroups from the “standard population,” which represented Taiwan’s total population on July 1, 2015, as officially announced by the Department of Household Registration, Ministry of the Interior [32]. Values of incidence, prevalence, and all-cause mortality rates were presented as numbers per 100,000 standard population. Additionally, we also calculated standardized IPF-specific all-cause mortality rates, presented in percentages. These rates were defined as the number of all-cause mortal cases in a study year divided by the total number of IPF patients who were alive in that same year, and then adjusted for sex- and age-subgroups within the standard population. Furthermore, we determined annual rates for specific treatments (medications or procedures) separately by dividing the total number of IPF patients receiving one specific treatment during a study year by the total number of IPF patients who were alive in that same year. We conducted statistical comparisons between groups using Fischer’s exact test or the Kruskal–Wallis test, as appropriate, to detect non-random differences. Longitudinal trends were assessed using the Mann–Kendall test and Theil-Sen estimator. All *p* values were two-sided, and statistical significance was set at *p* < 0.05. Our statistical analyses were performed using SAS version 9.4 (SAS Institute Inc., Cary, NC, USA), R version 3.6.3 (the R Foundation), and MedCal version 22.0.2 (MedCal Software, Belgium).

## Results

**Fig. 1:**
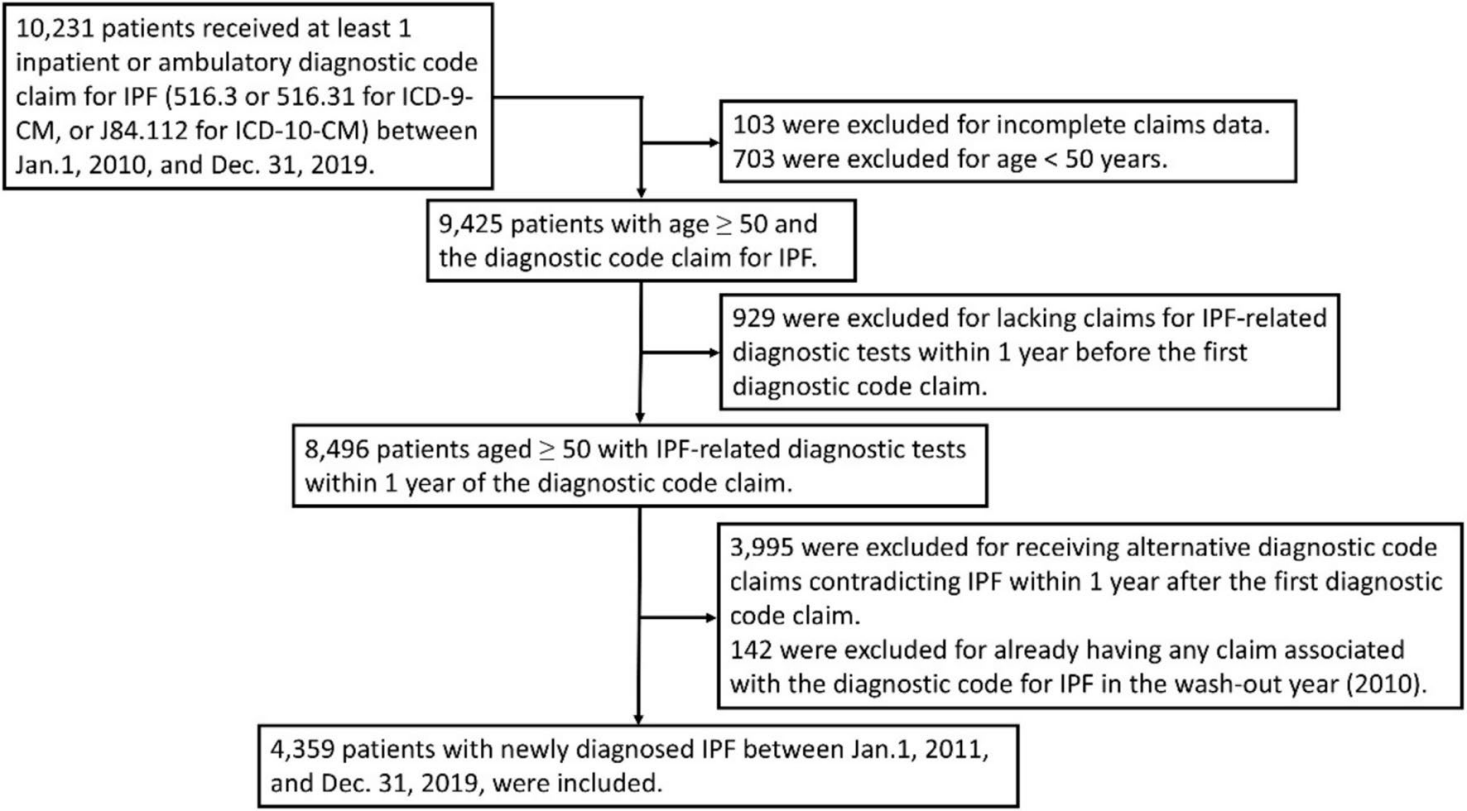
The flowchart of inclusion and exclusion of the present study

Between January 1, 2011, and December 31, 2019, a total of 4359 patients with newly diagnosed IPF were included (Fig. 1).

### Changing Trends in the Occurrence of IPF

**Fig. 2:**
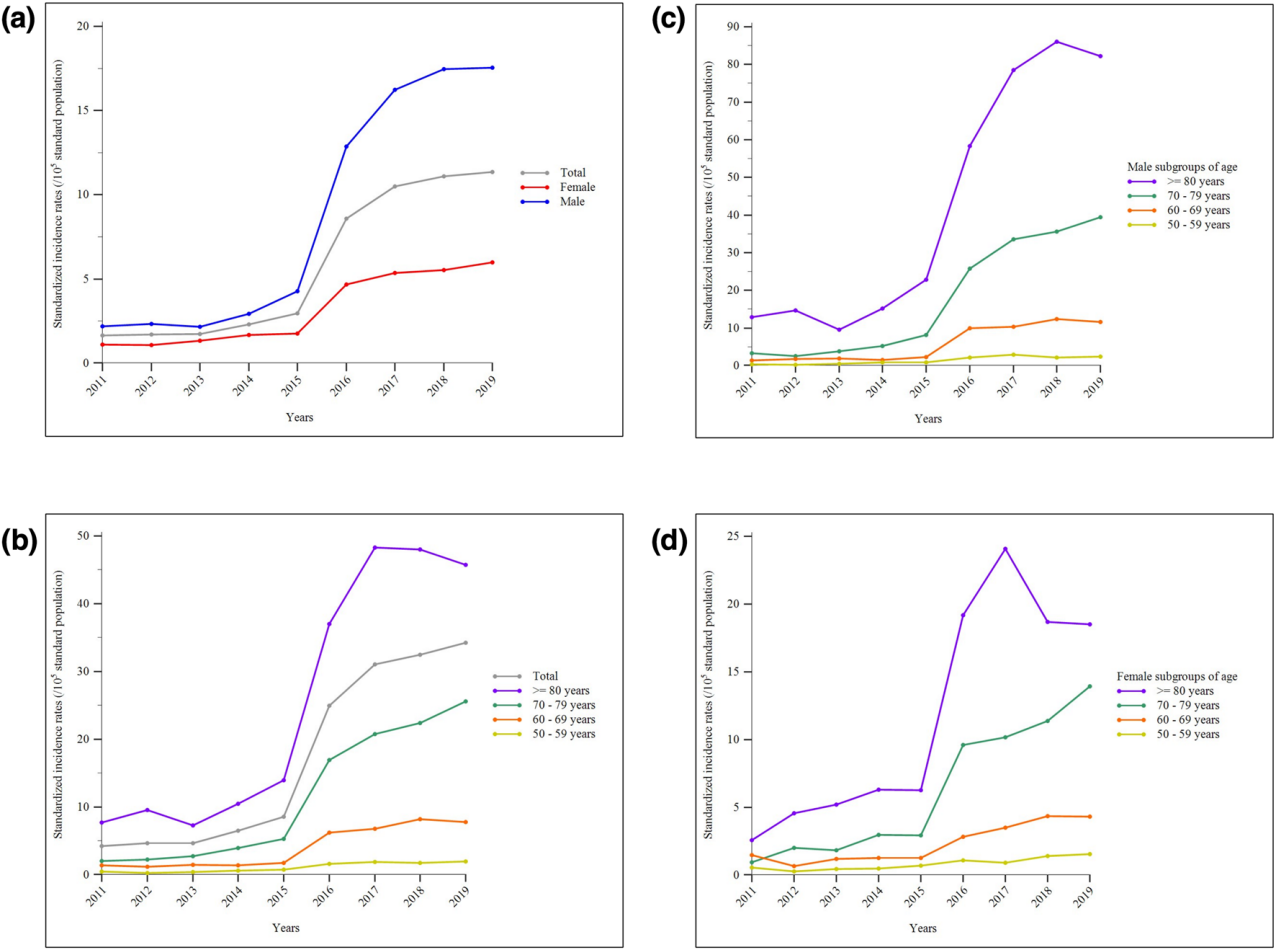
Standardized incidence rates of idiopathic pulmonary fibrosis in Taiwan between 2011 and 2019: **a** the whole population and sex-subgroups; **b** the whole population and age-subgroups; **c** age-subgroups in the male population; **d** age-subgroups in the female population

**Fig. 3:**
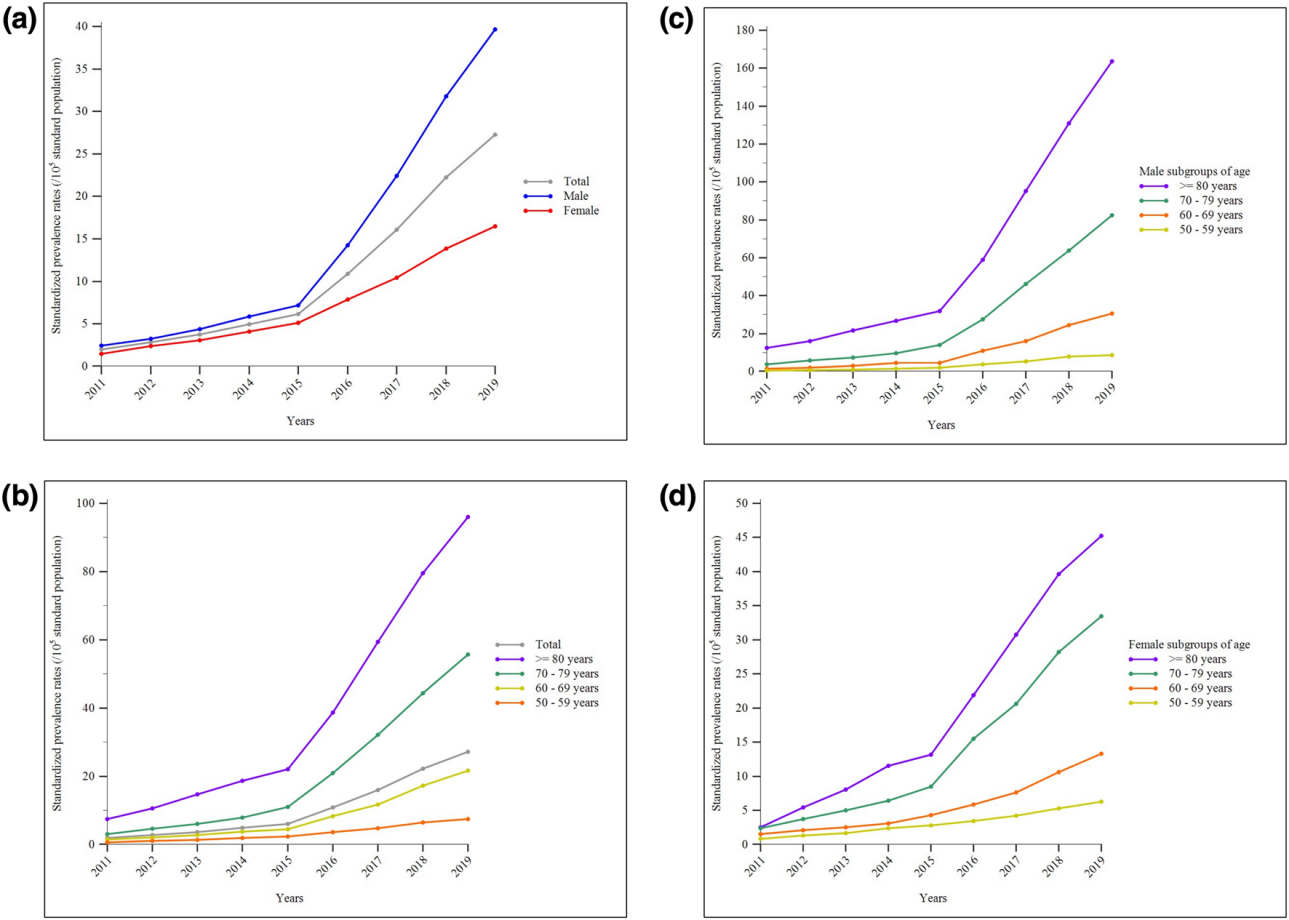
Standardized prevalence rates of idiopathic pulmonary fibrosis in Taiwan between 2011 and 2019: **a** the whole population and sex-subgroups; **b** the whole population and age-subgroups; **c** age-subgroups in the male population; **d** age-subgroups in the female population

During the study period, the mean standardized incidence rates of IPF in Taiwan were 6.09 (95% CI, 5.91–6.27), 9.04 (95% CI, 8.72–9.36), and 3.35 (95% CI, 3.17–3.54) cases per 100,000 standard population for the overall, male, and female populations, respectively. The annual crude and standardized incidence rates increased during the study period, exhibiting a sharply accelerated rise after 2015 for the entire population and across sex- and age-subgroups. Males consistently had a higher IPF diagnosis rate than females each year. Additionally, regardless of sex, the annual incidence rates were generally higher in older-age subgroups compared to consecutively younger ones (Supplemental Tables 3–4 and 5–6 show values of crude and standardized rates, respectively; Fig. 2A–D display changing trends of standardized rates; Supplemental Tables 7 show results of Mann–Kendall test and Theil-Sen estimator). Regarding IPF prevalence rates, the mean standardized values over the same period were 11.34 (95% CI, 11.17–11.52), 15.34 (95% CI, 14.92–15.75), and 7.64 (95% CI, 7.36–7.92) cases per 100,000 standard population for the overall, male, and female populations, respectively. Similar to incidence rates, the annual crude and standardized prevalence rates increased over the years and exhibited a sharp rise after 2015 for the entire population and across all sex- and age-subgroups. Male and older individuals consistently had higher annual prevalence rates than female and younger individuals (Supplemental Tables 8–9 and 10–11 show values of crude and standardized rates, respectively; Fig. 3A–D display changing trends of standardized rates; Supplemental Tables 7 shows results of Mann–Kendall test and Theil–Sen estimator).

### Mortality and Causes of Death

**Fig. 4:**
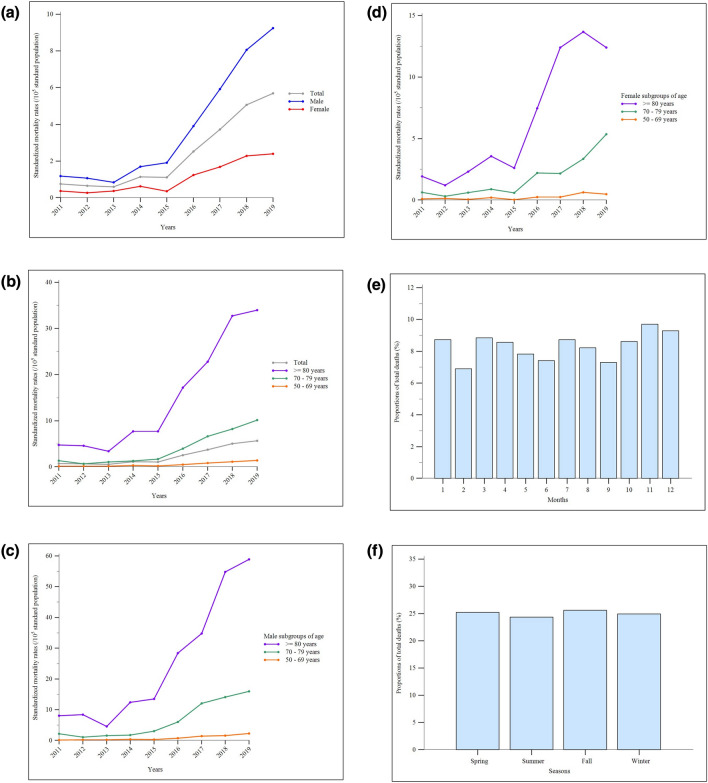

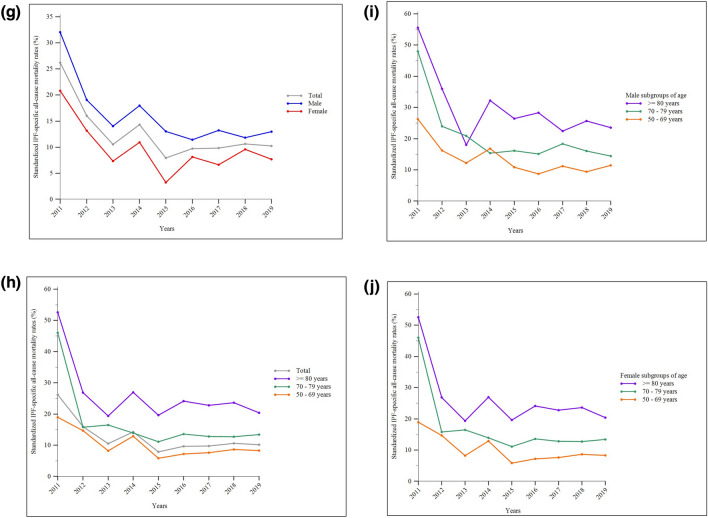
Standardized all-cause mortality rates of idiopathic pulmonary fibrosis (IPF) in Taiwan between 2011 and 2019: **a** the whole population and sex-subgroups; **b** the whole population and age-subgroups; **c** age-subgroups in the male population; **d** age-subgroups in the female population. **e** Monthly and **f** seasonal distribution of mortal cases (the *p* values for the Kruskal–Wallis test on the monthly and the seasonal distribution were 0.996 and 0.956, respectively). Standardized IPF-specific all-cause mortality rates over the same study period: **g** the whole population and sex-subgroups; **h** the whole population and age-subgroups; **i** age-subgroups in the male population; **j** age-subgroups in the female population

**Fig. 5:**
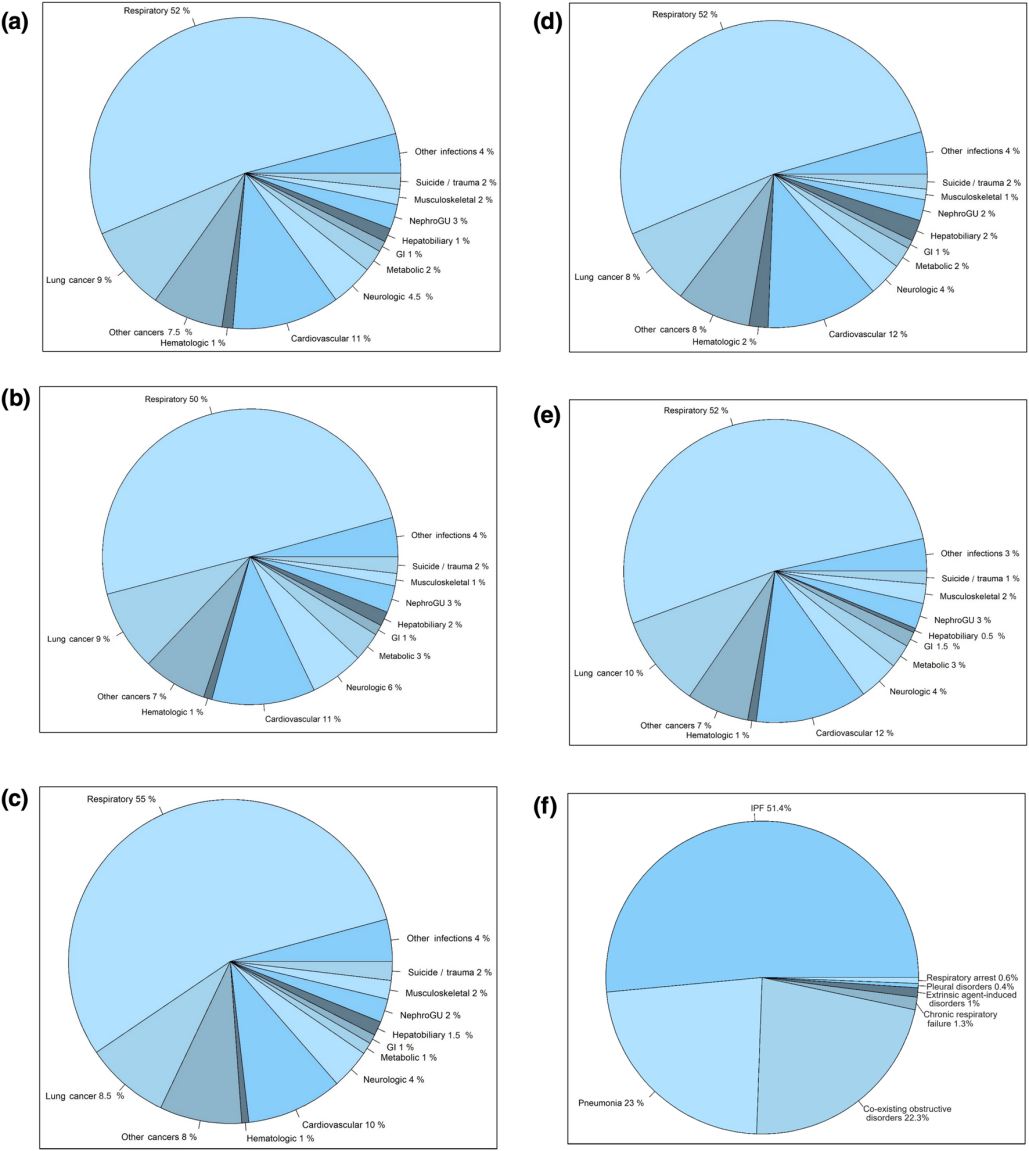
Causes of death of patients with idiopathic pulmonary fibrosis in Taiwan between 2011 and 2019: **a** overall distribution; **b** in spring; **c** in summer; **d** in fall; **e** in winter. **f** Distribution of specific non-neoplastic respiratory etiologies of death. Abbreviation: GI, gastrointestinal; IPF, idiopathic pulmonary fibrosis; NephroGU, nephrological and genitourinary

The mean standardized all-cause mortality rates for IPF in Taiwan during the study period were 2.36 (95% CI, 1.37–3.36), 3.76 (95% CI, 2.88–4.63), and 1.07 (95% CI, 0.59–1.55) per 100,000 standard population for the overall, male, and female populations, respectively. Annual mortality rates were generally higher among male and older individuals compared to female and younger individuals. There was no significant difference in the seasonal distribution of mortal cases. Between 2011 and 2015, the annual total and male standardized mortality rates exhibited progressively increasing trends, while the female standardized mortality rates fluctuated. Starting from 2015, there was an accelerated rise in standardized mortality rates across various sex- and age-subgroups, particularly among older-age subgroups (Fig. 4A–F and Supplemental Tables 7 and 12). In contrast, standardized IPF-specific all-cause mortality rates remained relatively stable in all subgroups after 2012 (Fig. 4G–J and Supplemental Tables 7 and 13). The leading cause of death were non-neoplastic respiratory etiologies, followed consecutively by cardiovascular disorders and lung cancer. This ranking remained consistent across the four seasons (Fig. 5A–E and Supplemental Table 14). Notably, progression of IPF was the most common non-neoplastic respiratory cause of death, accounting for over 50% of cases (Fig. 5F).

### Pharmacological Treatments

**Fig. 6:**
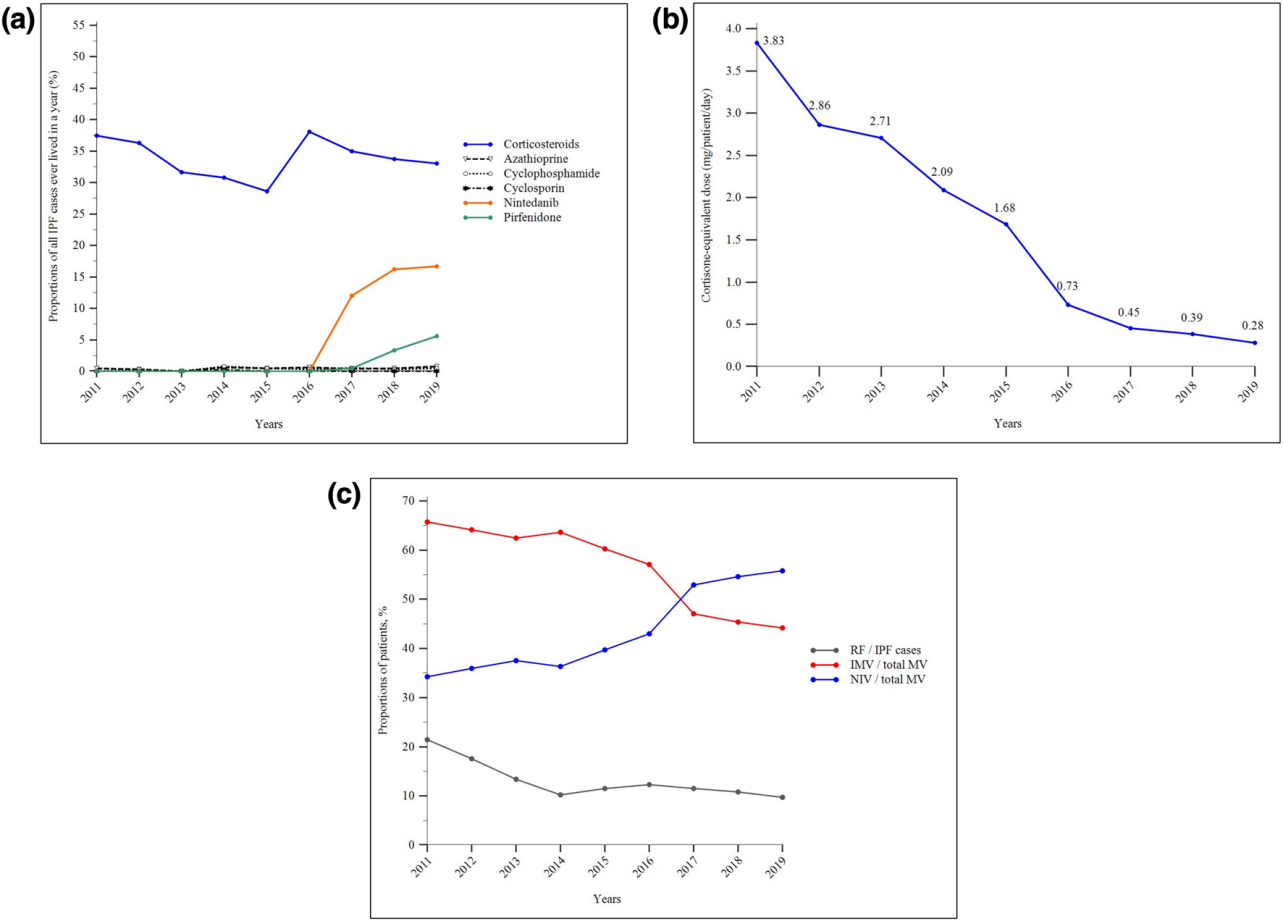
Shifting trends in the management of idiopathic pulmonary fibrosis in Taiwan between 2011 and 2019: **a** annual proportions of patients receiving at least one prescription of selected pharmacological therapies; **b** mean daily cortisone-equivalent dosage per patient every year; **c** annual rates of respiratory failure and the annual proportions of patients receiving invasive or non-invasive mechanical ventilation over all case of mechanical ventilation use. Abbreviations: IPF, idiopathic pulmonary fibrosis; IMV, invasive mechanical ventilation; MV, mechanical ventilation; NIV, non-invasive mechanical ventilation; RF, respiratory failure

During the study period, the annual proportions of Taiwan’s IPF patients receiving at least one prescription of azathioprine, cyclophosphamide, or cyclosporine were consistently very low (all below 0.8%). Approximately 30% of patients with IPF received at least one prescription of systemic corticosteroids each year. However, when all the prescriptions of corticosteroids each year were transformed into mean daily cortisone-equivalent dosages per patient, we observed a steady and significant declining trend in the mean daily dosage from 2011 through 2019. Moreover, a substantial rise in the annual proportions of patients receiving antifibrotic nintedanib and pirfenidone was observed after 2017, reaching 16.7% and 5.6%, respectively, by the end of 2019 (Fig. 6A–B and Supplemental Tables 15 and 16).

### Use of Mechanical Ventilation (MV)

The annual rates of respiratory failure remained relatively stable during the study period. Among all the patients with IPF receiving MV, the annual proportions of non-invasive MV (NIV) steadily increased, whereas the annual proportions of invasive MV (IMV) steadily declined. These trends led to a crossover point between 2016 and 2017 (Fig. 6C and Supplemental Tables 16 and 17).

## Discussion

In this study, we identified changing trends in the standardized incidence, prevalence, overall mortality and IPF-specific all-cause mortality rates of IPF in Taiwan from 2011 to 2019. We also demonstrated temporal changes in the pharmacological management patterns and the selection of mechanical ventilation modes during episode of respiratory failure among Taiwan’s IPF patients. Notably, our study period coincided with a time of major developments and modifications to international IPF guidelines. The accelerated rise in incidence and prevalence rates closely followed the 2015 therapeutic updates to international guidelines, which conditionally recommended the use of antifibrotic agents for the first time, and the initial introduction of Taiwan’s local IPF guideline. Consequently, our findings may demonstrate the longitudinal impact of evolving guidelines on the clinical landscape of IPF in Taiwan.

As the population composition of Taiwan remained stable throughout the study period, the difference between crude and standardized rates of incidence and prevalence was very small. The overall mean incidence and prevalence rates were higher than those reported by Taiwan’s previous researchers [18, 19] and were close to the values reported by Raghu et al. in 2006, using the “narrow definition” and a large database of healthcare claims in the US [28]. Our reported mean incidence and prevalence rates were also higher than those found in studies from Japan [22], South Korea [25], and several European countries (such as Finland, France, Greece) and were lower than those reported from Australia, Italy, the UK, Canada, and the older-age (≥ 65) population of the US [14, 15, 17, 26]. Our findings of the rising incidence and prevalence rates of IPF over time in Taiwan, on the other hand, align with earlier local and international epidemiological reports [14–25]. All reported cases of IPF in Taiwan were sporadic, with aging and cigarette smoking identified as important risk factors [1, 3, 6, 7]. Smoking could not account for our findings. There is generally a long period of cigarette smoke exposure before the development of chronic lung disease like IPF. Importantly, the proportion of smokers in Taiwan has declined steadily since 1990 [33]. The aging population might have contributed to the rising occurrence of IPF, given that Taiwan has transitioned into an “aged society” over the past two decades. Yet, this demographic shift alone could not sufficiently explain the epidemiological changes observed within just nine years, given that we also performed sex- and age-standardization of the incidence and prevalence rates. Enhanced awareness of IPF among both medical professionals and the general public is, we believe, a crucial factor, particularly for the dramatic rise in incidence and prevalence rates after 2015. This rise does not indicate that more people in Taiwan developed IPF, but rather that more patients who already had IPF received the correct diagnosis. The availability of novel antifibrotic agents since the mid-2010s for a previously untreatable disease like IPF was an impressive development. The management and the benefit standards of the NHI for IPF in Taiwan have generally adhered to evidence-based practices and international guidelines. Recent updates to the international guidelines, along with the publication of Taiwan’s local practice guidelines in the native language since 2015, expanded the scope of benefit of the NHI (e.g., coverage for antifibrotic therapy). These updates were also disseminated through various educational media and channels, thanks to collaborative efforts by public health officials and medical societies. All these have potentially contributed to drawing public attention to IPF, and sustaining this attention. The recent surge in the number of electronic news articles relating to “pulmonary fibrosis” from Taiwan’s top three news agencies (Supplemental Fig. 1) supports this heightened public awareness, potentially leading to improved disease vigilance and detection.

Consistent with previous reports from various regions [15, 34–37], our study demonstrated that the annual standardized all-cause mortality rates among Taiwan’s IPF patients increased across sex- and age-subgroups during the study period, approximately mirroring the trajectories observed for prevalence rates. Meanwhile, the annual standardized IPF-specific all-cause mortality rates and the rates of respiratory failure remained relatively stable. This apparent contradiction, in our view, could be attributed to the growing detection of new IPF cases. At a nationwide level, the prognosis of patients with IPF has not necessarily worsened over the study period, as the proportions of IPF patients dying from any cause did not significantly increase. Rather, heightened diagnostic vigilance has led to the identification of more patients for whom IPF was a major or relevant cause of death [38], resulting in larger numerators when calculating overall mortality rates. Similar to large-scale reports from Japan [21] and the United States [36, 37], non-neoplastic respiratory conditions ranked as the primary cause of death. Among these fatal respiratory conditions, IPF progression was the most common. Notably, acute exacerbation of IPF (AE-IPF) is catastrophic and an important cause of death [1, 21, 39]. Since AE-IPF occurs more frequently in the advanced stage, presenting similarly to severe pneumonia and lacking a specific ICD code, some mortal cases classified as IPF progression or pneumonia-related death might actually be due to AE-IPF. Furthermore, although seasonal spikes in air pollution and respiratory viral infections pose risks to IPF patients [40], we found no significant difference in the seasonal distribution of mortality cases and causes.

The patterns of pharmacological management for IPF in Taiwan between 2011 and 2019, as revealed by our study, aligned with the recent evolution of guideline recommendations, which has increasingly discouraged immunosuppression while supporting antifibrotic therapy [2, 3, 5, 7]. However, it is important to acknowledge that each year, some patients still received prescriptions for systemic corticosteroid (although the mean daily dosages evidently decreased) or nonsteroidal immunosuppressants. Several factors may explain these therapeutic practices. Firstly, corticosteroids are commonly used for various medical conditions, and some of these patients might have received systemic corticosteroids for non-IPF indications. Secondly, although guidelines have recommended against routine immunosuppressant use as maintenance therapy for IPF since 2011, there is as yet no standardized treatment for AE-IPF. Systemic corticosteroid remains an acceptable option [3, 7, 41], and there have been reports of using nonsteroidal immunosuppressants for AE-IPF [3, 41]. Findings from the EXAFIP trial, which questioned the efficacy of cyclophosphamide in AE-IPF, were published only recently [42]. Furthermore, antifibrotic therapy is costly and may pose a significant financial burden for ordinary households in Taiwan. Although the NHI has covered antifibrotic therapy expense for IPF since 2017, the initial reimbursement criteria were relatively strict. This accounted for the fact that, despite a noticeable increase, only a minority of patients received antifibrotic therapy by the end of 2019.

There are limitations to our present study. The WPD of Taiwan’s NHI allowed us to gain a nationwide overview of longitudinal epidemiological and therapeutic trends related to IPF. However, the WPD lacked specific details such as individual medical records, spirometric readings, and computed tomographic findings. Our definition of IPF had to rely on code-based criteria rather than actual diagnostic criteria [12]. To mitigate potential bias from miscoding and misclassification, we refined the definition by incorporating three additional necessary conditions. We also independently validated the accuracy of the definition we used. Nevertheless, the possibility of misclassification may still exist. While our working definition of IPF demonstrated good performance in the validation cohort (comprising patients from our hospital, a tertiary medical center and a regional ILD referral center), this may not necessarily ensure the same accuracy in the WPD. Given the stringent criteria of our working definition, we might have underestimated the true occurrence of IPF in Taiwan. Secondly, the data we present regarding pharmacological management were based on insurance claims for medicinal prescriptions declared by clinicians at NHI-contracted institutes. The WPD did not contain information about patient compliance. However, it is important to note that our analysis primarily focused on the changing patterns of medical practices. Therefore, the lack of patient compliance data should not significantly impact our findings in this context. Thirdly, we were unable to include analyses of medical treatments covered by private insurances or those paid for directly by patients. Nevertheless, given the near-universal coverage of Taiwan’s NHI throughout the study period, we believe this limitation should not substantially affect our overall conclusions.

## Conclusion

Between 2011 and 2019, the epidemiology and management of IPF in Taiwan exhibited shifting trends, with increasing incidence and prevalence rates. IPF predominantly affected males and older individuals, with higher mortality rates among these groups. Most mortal cases were due to IPF progression. Over time, there were significant changes in treatments, with decreased use of immunosuppressants, increased use of antifibrotic agents, and a preference for NIV over IMV. These trends reflect the impact of evolving IPF guidelines on clinical practices. Our findings contribute valuable snapshots from the East Asia region to the global epidemiological panorama of IPF. They also highlight the importance of clinical vigilance in enhancing the diagnostic detection of IPF, thereby revealing the true burden of the disease and initiating timely treatment to slow its progression.

## Supplementary Information

Below is the link to the electronic supplementary material.

Supplementary file1 (PDF 1225 KB)

## Data Availability

Data from the Whole Population Datafiles of the National Health Insurance Research Database (NHIRD): data may be obtained from a third party and are not publicly available. The data which were interpreted in the present study were applied restrictively from the NHIRD, so that this database was used under license limited to the study. In addition, the data are not publicly available. The NHIRD is published by National Health Insurance Administration of Taiwan, in compliance with Taiwan’s “Personal Information Protection Act”. Requests for data can be submitted as a formal application to the NHIRD (https://dep.mohw.gov.tw/DOS/lp-2503-113.html). Data of the validation cohort: the de-identified datasets used and analysed to validate the IPF definition for the current study are available from the corresponding author on reasonable request.

## Abbreviations

AE-IPF: Acute exacerbations of idiopathic pulmonary fibrosis
CI: Confidence interval
COPD: Chronic obstructive pulmonary disease
GI: Gastrointestinal
ICD-9/-10-CM: International classification of disease, nineth or tenth revision, clinical modification
ILD: Interstitial lung disease
IMV: Invasive mechanical ventilation
IPF: Idiopathic pulmonary fibrosis
MV: Mechanical ventilation
NephroGU: Nephrological and genitourinary
NHI: National health insurance
NIV: Non-invasive mechanical ventilation
RF: Respiratory failure
WPD: Whole-population datafiles

## References

[1] Lederer DJ, Martinez FJ. Idiopathic pulmonary fibrosis. N Engl J Med. 2018;378(19):1811–23. 29742380 10.1056/NEJMra1705751

[2] American Thoracic Society; European Respiratory Society. Idiopathic pulmonary fibrosis: diagnosis and treatment: international consensus statement. Am J Respir Crit Care Med. 2000;161:646–64. 10673212 10.1164/ajrccm.161.2.ats3-00

[3] Raghu G, Collard HR, Egan JJ, et al. ATS/ERS/JRS/ALAT committee on idiopathic pulmonary fibrosis. An official ATS/ERS/JRS/ALAT statement: idiopathic pulmonary fibrosis: evidence-based guidelines for diagnosis and management. Am J Respir Crit Care Med. 2011;183:788–824. 21471066 10.1164/rccm.2009-040GL PMC5450933

[4] Travis WD, Costabel U, Hansell DM, et al. An official American Thoracic Society/European Respiratory Society statement: update of the international multidisciplinary classification of the idiopathic interstitial pneumonias. Am J Respir Crit Care Med. 2013;188(6):733–48. 24032382 10.1164/rccm.201308-1483ST PMC5803655

[5] Raghu G, Rochwerg B, Zhang Y, et al., American Thoracic Society, European Respiratory society, Japanese Respiratory Society, Latin American Thoracic Association. An official ATS/ERS/JRS/ALAT clinical practice guideline: treatment of idiopathic pulmonary fibrosis. an update of the 2011 Clinical practice guideline. Am J Respir Crit Care Med. 2015;192(2):e3–e19. 10.1164/rccm.201506-1063ST 26177183

[6] Raghu G, Remy-Jardin M, Myers JL, et al., American Thoracic Society, European Respiratory Society, Japanese Respiratory Society, and Latin American Thoracic Society. Diagnosis of idiopathic pulmonary fibrosis an official ATS/ERS/JRS/ALAT clinical practice guideline. Am J Respir Crit Care Med. 2018;198(5):e44–e68. 10.1164/rccm.201807-1255ST 30168753

[7] Raghu G, Remy-Jardin M, Richeldi L, et al. Idiopathic pulmonary fibrosis (an update) and progressive pulmonary fibrosis in adults: an official ATS/ERS/JRS/ALAT clinical practice guideline. Am J Respir Crit Care Med. 2022;205(9):e18–47. 35486072 10.1164/rccm.202202-0399ST PMC9851481

[8] Wang HC, Yu CJ, Lee CH, et al. Evidence-based guidelines for diagnosis and management of idiopathic pulmonary fibrosis. 1st ed. Taipei: Taiwan Society of Pulmonary and Critical Care Medicine; 2015.

[9] Cottin V, Cadranel J, Crestani B, et al. Management of idiopathic pulmonary fibrosis in France: a survey of 1244 pulmonologists. Respir Med. 2014;108(1):195–202. 24361163 10.1016/j.rmed.2013.11.017

[10] Cottin V, Bergot E, Bourdin A, et al. Adherence to guidelines in idiopathic pulmonary fibrosis: a follow-up national survey. ERJ Open Res. 2015;1(2):00032–2015. 27730153 10.1183/23120541.00032-2015 PMC5005118

[11] Munson JC, Kreider M, Chen Z, et al. Effect of treatment guidelines on the initial management of idiopathic pulmonary fibrosis. Br J Clin Pharmacol. 2010;70(1):118–25. 20642554 10.1111/j.1365-2125.2010.03670.x PMC2909814

[12] Caminati A, Madotto F, Cesana G, et al. Epidemiological studies in idiopathic pulmonary fibrosis: pitfalls in methodologies and data interpretation. Eur Respir Rev. 2015;24(137):436–44. 26324805 10.1183/16000617.0040-2015 PMC9487686

[13] Behr J, Kreuter M, Hoeper MM, et al. Management of patients with idiopathic pulmonary fibrosis in clinical practice: the INSIGHTS-IPF registry. Eur Respir J. 2015;46(1):186–96. 25837040 10.1183/09031936.00217614 PMC4486374

[14] Ley B, Collard HR. Epidemiology of idiopathic pulmonary fibrosis. Clin Epidemiol. 2013;5:483–92. 24348069 10.2147/CLEP.S54815 PMC3848422

[15] Hutchinson J, Fogarty A, Hubbard R, et al. Global incidence and mortality of idiopathic pulmonary fibrosis: a systematic review. Eur Respir J. 2015;46(3):795–806. 25976683 10.1183/09031936.00185114

[16] Sauleda J, Núñez B, Sala E, et al. Idiopathic pulmonary fibrosis: epidemiology, natural history, phenotypes. Med Sci (Basel). 2018;6(4):110. 30501130 10.3390/medsci6040110 PMC6313500

[17] Maher TM, Bendstrup E, Dron L, et al. Global incidence and prevalence of idiopathic pulmonary fibrosis. Respir Res. 2021;22(1):197. 34233665 10.1186/s12931-021-01791-z PMC8261998

[18] Lai CC, Wang CY, Lu HM, et al. Idiopathic pulmonary fibrosis in Taiwan—a population-based study. Respir Med. 2012;106(11):1566–74. 22954482 10.1016/j.rmed.2012.07.012

[19] Yang SN, Perng DW, Ko HK, et al. Epidemiologic analysis of Taiwanese patients with idiopathic pulmonary fibrosis. Healthcare (Basel). 2020;8(4):580. 33371337 10.3390/healthcare8040580 PMC7767390

[20] Huang H, Peng X, Zhong C. Idiopathic pulmonary fibrosis: the current status of its epidemiology, diagnosis, and treatment in China. Intractable Rare Dis Res. 2013;2(3):88–93. 25343109 10.5582/irdr.2013.v2.3.88 PMC4204549

[21] Ohno S, Nakaya T, Bando M, et al. Idiopathic pulmonary fibrosis–results from a Japanese nationwide epidemiological survey using individual clinical records. Respirology. 2008;13(6):926–8. 18657060 10.1111/j.1440-1843.2008.01349.x

[22] Natsuizaka M, Chiba H, Kuronuma K, et al. Epidemiologic survey of Japanese patients with idiopathic pulmonary fibrosis and investigation of ethnic differences. Am J Respir Crit Care Med. 2014;190:773–9. 25162152 10.1164/rccm.201403-0566OC

[23] Gjonbrataj J, Choi WI, Bahn YE, et al. Incidence of idiopathic pulmonary fibrosis in Korea based on the 2011 ATS/ERS/JRS/ALAT statement. Int J Tuberc Lung Dis. 2015;19(6):742–6. 25946370 10.5588/ijtld.14.0650

[24] Han S, Mok Y, Jee SH, et al. Incidence and mortality of idiopathic pulmonary fibrosis in South Korea. ATS Am J Respir Crit Care Med. 2013;187:A1460.

[25] Joung KI, Park H, Park S, et al. Nationwide epidemiologic study for fibrosing interstitial lung disease (F-ILD) in South Korea: a population-based study. BMC Pulm Med. 2023;23(1):98. 36949407 10.1186/s12890-023-02373-z PMC10035232

[26] Cox IA, Otahal P, de Graaff B, et al. Incidence, prevalence and mortality of idiopathic pulmonary fibrosis in Australia. Respirology. 2022;27(3):209–16. 34935240 10.1111/resp.14194

[27] Annual coverage rates of the National Health Insurance. Gender Equality Committee, Executive Yuan of Taiwan, R.O.C. Updated February 23, 2024. Accessed 4 Aug 2024. https://www.gender.ey.gov.tw/gecdb/Stat_Statistics_Query.aspx?sn=Qz3BX0VivH9e05eqU64qzw%40%40&statsn=u4ceyDJ9iGzBYUGlJC0z7w%40%40

[28] Raghu G, Weycker D, Edelsberg J, et al. Incidence and prevalence of idiopathic pulmonary fibrosis. Am J Respir Crit Care Med. 2006;174(7):810–6. 16809633 10.1164/rccm.200602-163OC

[29] Ley B, Urbania T, Husson G, et al. Code-based diagnostic algorithms for idiopathic pulmonary fibrosis. Case validation and improvement. Ann Am Thorac Soc. 2017;14(6):880–7. 28355518 10.1513/AnnalsATS.201610-764OC PMC5566307

[30] Moua T, Maldonado F, Decker PA, et al. Frequency and implication of autoimmune serologies in idiopathic pulmonary fibrosis. Mayo Clin Proc. 2014;89(3):319–26. 24582190 10.1016/j.mayocp.2013.11.018

[31] Liu D, Ahmet A, Ward L, et al. A practical guide to the monitoring and management of the complications of systemic corticosteroid therapy. Allergy Asthma Clin Immunol. 2013;15(1):30. 10.1186/1710-1492-9-30 PMC3765115 23947590

[32] Standard population of Taiwan. Department of Household Registration, Ministry of the Interior of Taiwan, R.O.C. Updated December 31, 2023. Last accessed 15 Mar 2024. https://www.ris.gov.tw/app/portal/346

[33] Annual proportions of smokers in Taiwan. Health Promotion Administration, Department of Household Registration, Ministry of Health and Welfare of Taiwan, R.O.C. Updated June 25, 2024. Last accessed 1 Aug 2024. https://www.hpa.gov.tw/Pages/Detail.aspx?nodeid=1718&pid=9913

[34] Hutchinson JP, McKeever TM, Fogarty AW, et al. Increasing global mortality from idiopathic pulmonary fibrosis in the twenty-first century. Ann Am Thorac Soc. 2014;11(8):1176–85. 25165873 10.1513/AnnalsATS.201404-145OC

[35] Marshall DC, Salciccioli JD, Shea BS, et al. Trends in mortality from idiopathic pulmonary fibrosis in the European Union: an observational study of the WHO mortality database from 2001–2013. Eur Respir J. 2018;51(1):1701603. 29348182 10.1183/13993003.01603-2017

[36] Mannino DM, Etzel RA, Parrish RG. Pulmonary fibrosis deaths in the United States, 1979–1991: an analysis of multiple-cause mortality data. Am J Respir Crit Care Med. 1996;153:1548–52. 8630600 10.1164/ajrccm.153.5.8630600

[37] Olson AL, Swigris JJ, Lezotte DC, et al. Mortality from pulmonary fibrosis increased in the United States from 1992 to 2003. Am J Respir Crit Care Med. 2007;176(3):277–84. 17478620 10.1164/rccm.200701-044OC

[38] Ryerson CJ, Kolb M. The increasing mortality of idiopathic pulmonary fibrosis: fact or fallacy? Eur Respir J. 2018;51(1):1702420. 29348187 10.1183/13993003.02420-2017

[39] Huang TH, Wei SH, Kuo HI, et al. Baseline blood levels of mucin-1 are associated with crucial on-treatment adverse outcomes in patients with idiopathic pulmonary fibrosis receiving antifibrotic pirfenidone. Biomedicines. 2024;12(2):402. 38398004 10.3390/biomedicines12020402 PMC10886731

[40] Ho ATN, Shmelev A, Charbek E. Trends and seasonal variation of hospitalization and mortality of interstitial lung disease in the United States from 2006 to 2016. Respir Res. 2020;21(1):152. 32546158 10.1186/s12931-020-01421-0 PMC7298940

[41] Juarez MM, Chan AL, Norris AG, et al. Acute exacerbation of idiopathic pulmonary fibrosis-a review of current and novel pharmacotherapies. J Thorac Dis. 2015;7(3):499–519. 25922733 10.3978/j.issn.2072-1439.2015.01.17 PMC4387423

[42] Naccache JM, Jouneau S, Didier M, et al., EXAFIP investigators and the OrphaLung network. Cyclophosphamide added to glucocorticoids in acute exacerbation of idiopathic pulmonary fibrosis (EXAFIP): a randomised, double-blind, placebo-controlled, phase 3 trial. Lancet Respir Med. 2022;10(1):26–34. 10.1016/S2213-2600(21)00354-4 34506761

