# Supplementary material for: Shifting Trends in the Epidemiology and Management of Idiopathic Pulmonary Fibrosis in the Era of Evidence-Based Guidelines: a Nationwide Population Study
Source: J Epidemiol Glob Health. 2025 Mar 17;15(1):44. doi: 10.1007/s44197-025-00377-y (PMC11914588; doi:10.1007/s44197-025-00377-y)
Supplement: Supplementary file 1 — Supplementary file1 (PDF 1225 KB) [file 44197_2025_377_MOESM1_ESM.pdf]

Title: Shifting trends in the epidemiology and management of idiopathic pulmonary fibrosis in the era of evidence-based guidelines: a nationwide population study

Authors: Tang-Hsiu Huang, Shen-Huan Wei, Chin-Wei Kuo, Hsin-Yu Hou, Chao-Liang Wu, Sheng-Hsiang Lin

Online Supplemental Materials

Corresponding author details:

Sheng-Hsiang Lin, Ph.D.

Institute of Clinical Medicine, College of Medicine, National Cheng Kung University, 35 Siaodong Rd., Tainan 70457, Tainan, Taiwan

Email: shlin922@mail.ncku.edu.tw

Table of Content

Supplemental Material	Page
**Supplemental Table 1** Procedural codes of Taiwan's National Health Insurance (NHI) used in the present study	3
**Supplemental Table 2** International Classification of Diseases, 9th and 10th Revision, Clinical Modification codes of disease entities/conditions that contradict the diagnosis of idiopathic pulmonary fibrosis in the present study	4
**Supplemental Table 3** Annual incidence cases and crude incidence rates of idiopathic pulmonary fibrosis in Taiwan between 2011 and 2019	5
**Supplemental Table 4** Annual incidence cases and crude incidence rates of idiopathic pulmonary fibrosis of sex-specific age subgroups in Taiwan between 2011 and 2019	6
**Supplemental Table 5** Annual incidence cases and standardized incidence rates of idiopathic pulmonary fibrosis in Taiwan between 2011 and 2019	7
**Supplemental Table 6** Annual incidence cases and standardized incidence rates of idiopathic pulmonary fibrosis of sex-specific age subgroups in Taiwan between 2011 and 2019	8
**Supplemental Table 7** Summary of results from the Mann-Kendall test and Theil-Sen estimator on longitudinal epidemiological trends of patients with idiopathic pulmonary fibrosis in Taiwan between 2011 and 2019	9
**Supplemental Table 8** Annual prevalence cases and crude prevalence rates of idiopathic pulmonary fibrosis in Taiwan between 2011 and 2019	10
**Supplemental Table 9** Annual prevalence cases and crude prevalence rates of idiopathic pulmonary fibrosis of sex-specific age subgroups in Taiwan between 2011 and 2019	11
**Supplemental Table 10** Annual prevalence cases and standardized prevalence rates of idiopathic pulmonary fibrosis in Taiwan between 2011 and 2019	12
**Supplemental Table 11** Annual prevalence cases and standardized prevalence rates of idiopathic pulmonary fibrosis of sex-specific age subgroups in Taiwan between 2011 and 2019	13
**Supplemental Table 12** Annual mortal cases and standardized all-cause mortality rates of idiopathic pulmonary fibrosis of in Taiwan between 2011 and 2019	14
**Supplemental Table 13** Annual standardized IPF-specific all-cause mortality rates of patients with idiopathic pulmonary fibrosis in Taiwan between 2011 and 2019	15
**Supplemental Table 14** The overall and seasonal distribution of causes of death of patients with Idiopathic pulmonary fibrosis in Taiwan between 2011 and 2019	16
**Supplemental Table 15** Annual proportions of patients with idiopathic pulmonary fibrosis receiving at least one prescription of selected medications	17
**Supplemental Table 16** Summary of results from the Mann-Kendall test and Theil-Sen estimator on longitudinal trends of specific managements for patients with idiopathic pulmonary fibrosis in Taiwan between 2011 and 2019	18
**Supplemental Table 17** Annual proportions of respiratory failure and invasive and noninvasive mechanical ventilation	19
**Supplemental Figure 1** Annual numbers of electronic news articles relating to "pulmonary fibrosis" from Taiwan's top 3 news agencies between 2000 and 2020	20
**Appendix 1:** Validation process of the working definition of idiopathic pulmonary fibrosis (IPF) for this study	21-23

Supplemental Table 1 Procedural codes of Taiwan's National Health Insurance (NHI) used in the present study

NHI procedural code	Code definition
**Invasive studies**	
**NHI procedural code**	**Code definition**
15020B	Transbronchial biopsy
19007B	Ultrasonography-guided tissue biopsy
25004C	Level IV surgical pathology processing and interpretation
28006C	Bronchoscopy
28009B	Thoracoscopic examination with biopsy
28026B	Mediastinoscopic examination with biopsy
28030C	Endoscopic examination with biopsy
33103B	Computed tomography (CT)-guided tissue biopsy
51027B, 51028B	Excisional biopsy of tissue
67051B	Thoracoscopic wedge or partial resection of the Lung
92021B, 92022B	Biopsy of tissue
92067B, 92068B	Incisional biopsy of tissue
**Imaging studies**	
**NHI procedural code**	**Code definition**
33070B	Computed tomography without contrast medium use
33071B	Computed tomography with contrast medium use
33072B	Computed tomography with and without contrast medium use
33090B	Computed tomography with the use of nonionic contrast medium
**Mechanical ventilation**	
**NHI procedural code**	**Code definition**
57001B, 47031C	Invasive mechanical ventilation
57023B	Non-invasive mechanical ventilation

The procedural codes and their definitions are officially issued by Taiwan's National Health Insurance Administration, Ministry of Health and Welfare, at: https://info.nhi.gov.tw/INAE5000/INAE5001S01.

Supplemental Table 2 International Classification of Diseases, 9th and 10th Revision, Clinical Modification codes of disease entities/conditions that contradict the diagnosis of idiopathic pulmonary fibrosis in the present study

Diagnosis	ICD-9-CM codes	ICD-10-CM codes
Pulmonary sarcoidosis	135	D86.0
Hypersensitivity pneumonia (HP)	495.9	J67.9
Pneumoconiosis/coal worker's lung	500	J60
Pneumoconiosis due to asbestos and other mineral fibers	501	J61
Pneumoconiosis due to talc or other dust containing silica	502	J62.0, J62.8
Pneumoconiosis due to various other inorganic dusts	503	J63.0 – J63.6
Pulmonary diseases due to inhalation of organic dusts	504	J66.0, J66.1, J66.2, J66.8
Pneumoconiosis, unspecified	505	J64, J65
Chronic respiratory conditions due to fumes and vapors	506.4	J68.4, J68.8
Chronic and other pulmonary manifestations due to radiation or other specified or unspecified external agent	508.0 - 508.9	J70.0 – J70.9
Post-inflammation fibrosis	515	J84.17, J84.89,
Pulmonary alveolar proteinosis (PAP)	516.0	J84.01
Idiopathic pulmonary hemosiderosis	516.1	J84.03
Pulmonary alveolar microlithiasis	516.2	J84.02
Cryptogenic organizing pneumonia (COP)	516.36	J84.116
Other alveolar and parieto-alveolar conditions		J84.09
Idiopathic non-specific interstitial pneumonia (iNSIP)		J84.111, J84.113
Acute interstitial pneumonia (AIP)		J84.114
Desquamative interstitial pneumonia (DIP)	516.8	J84.117
Lymphoid interstitial pneumonia (LIP)		J84.2
Other specified interstitial pulmonary diseases		J84.89
Other pulmonary collapse		J98.19
Interstitial pulmonary disease, unspecified	516.9	J84.9
Lung involvement in other diseases classified elsewhere	517.8	J99
Interstitial emphysema	518.1	J98.2
Pulmonary eosinophilia	518.3	J82
Systemic lupus erythematosus (SLE), with or without other organ involvement	710.0	M32.0, M32.10, M32.19
Systemic sclerosis, with or without lung involvement	517.2, 710.1	M34.0, M34.1, M34.89, M34.81, M34.9
Sicca syndrome/Sjogren syndrome, with or without other organ involvement	710.2	M35.00, M35.01, M35.09
Dermatomyositis, with or without other organ involvement	710.3	M33.00, M33.09, M33.10, M33.19, M33.90, M33.99, M36.0
Polymyositis, with or without other organ involvement	710.4	M33.20, M33.29
Overlapping syndromes of connective tissue diseases	710.8	M35.1, M35.5
Systemic involvement of connective tissue, unspecified	710.9	M35.9
Rheumatoid arthritis (RA) with or without lung involvement	517.1, 714.81	J17, M05.10, M05.111, M05.112, M05.119, M05.121, M05.122, M05.129, M05.131, M05.132, M05.139, M05.141, M05.142, M05.149, M05.151, M05.152, M05.159, M05.161, M05.162, M05.169, M05.171, M05.172, M05.179, M05.19
Ankylosing spondylitis (AS), with or without respiratory involvement	720.0	M08.1, M45.0 M45.1, M45.2, M45.3, M45.4, M45.5, M45.6, M45.7, M45.8, M45.9,
Tuberous sclerosis (TSC), with or without lung involvement	759.5	Q85.1

Supplemental Table 3 Annual **incidence cases** and **crude incidence rates** of idiopathic pulmonary fibrosis in Taiwan between 2011 and 2019

Year	Overall		Male		Female		Male to female ratios
	Cases	Rates[1]	Cases	Rates[1]	Cases	Rates[1]	
2011	114	1.63 (1.35 - 1.96)	76	2.23 (1.77 - 2.80)	38	1.06 (0.75 - 1.45)	2.11 (1.43 – 3.12)
2012	123	1.70 (1.41 - 2.03)	83	2.36 (1.88 - 2.93)	40	1.07 (0.77 - 1.46)	2.20 (1.51 – 3.21)
2013	130	1.74 (1.45 - 2.06)	79	2.18 (1.73 - 2.72)	51	1.32 (0.98 - 1.74)	1.65 (1.16 – 2.35)
2014	178	2.30 (1.98 - 2.67)	111	2.98 (2.45 - 3.59)	67	1.68 (1.30 - 2.13)	1.78 (1.31 – 2.41)
2015	237	2.98 (2.61 - 3.38)	164	4.29 (3.66 - 4.99)	73	1.77 (1.39 - 2.22)	2.42 (1.84 – 3.19)
2016	703	8.60 (7.97 - 9.26)	503	12.82 (11.73 - 13.99)	200	4.70 (4.07 - 5.40)	2.73 (2.32 – 3.21)
2017	883	10.53 (9.85 - 11.25)	645	16.07 (14.86 - 17.36)	238	5.44 (4.77 - 6.18)	2.95 (2.55 – 3.43)
2018	967	11.26 (10.56 - 11.99)	713	17.39 (16.14 - 18.71)	254	5.66 (4.98 - 6.40)	3.07 (2.66 – 3.55)
2019	1024	11.63 (10.93 - 12.37)	739	17.63 (16.38 - 18.95)	285	6.18 (5.49 - 6.94)	2.85 (2.49 – 3.27)
**Mean**	484	6.11 (5.93 - 6.29)	345	9.07 (8.76 - 9.40)	138	3.36 (3.18 - 3.56)	2.70 (2.53 – 2.88)

Year	Age group 50 - 59		Age group 60 - 69		Age group 70 - 79		Age group ≥ 80	
	Cases	Rates[1]	Cases	Rates[1]	Cases	Rates[1]	Cases	Rates[1]
2011	16	0.47 (0.27 - 0.77)	26	1.42 (0.93 - 2.08)	24	2.07 (1.33 - 3.09)	48	7.74 (5.71 - 10.27)
2012	10	0.29 (0.14 - 0.53)	24	1.22 (0.78 - 1.82)	27	2.29 (1.51 - 3.33)	62	9.58 (7.34 - 12.28)
2013	16	0.46 (0.26 - 0.74)	32	1.52 (1.04 - 2.15)	33	2.74 (1.88 - 3.84)	49	7.30 (5.40 - 9.65)
2014	24	0.67 (0.43 - 1.00)	32	1.43 (0.98 - 2.02)	49	3.99 (2.95 - 5.27)	73	10.53 (8.26 - 13.24)
2015	28	0.78 (0.52 - 1.12)	43	1.79 (1.29 - 2.41)	66	5.34 (4.13 - 6.79)	100	13.98 (11.38 - 17.01)
2016	59	1.63 (1.24 - 2.10)	162	6.27 (5.34 - 7.31)	210	16.94 (14.73 - 19.40)	272	36.99 (32.73 - 41.66)
2017	70	1.93 (1.50 - 2.44)	186	6.79 (5.85 - 7.84)	262	20.77 (18.33 - 23.45)	365	48.31 (43.48 - 53.53)
2018	65	1.79 (1.38 - 2.28)	235	8.20 (7.18 - 9.32)	293	22.42 (19.93 - 25.14)	374	48.04 (43.30 - 53.17)
2019	72	1.98 (1.55 - 2.49)	233	7.81 (6.84 - 8.88)	351	25.58 (22.97 - 28.40)	368	45.78 (41.22 - 50.70)
**Mean**	40	1.12 (1.01 - 1.25)	108	4.48 (4.20 - 4.77)	146	11.75 (11.12 - 12.40)	190	26.65 (25.40 - 27.94)

[1] Values indicate crude incidence rates (and the corresponding 95% confidence intervals in parentheses) per 100,000 persons of Taiwan's population on July 1 of each study year.

Supplemental Table 4 Annual **incidence cases** and **crude incidence rates** of idiopathic pulmonary fibrosis of **sex-specific age subgroups** in Taiwan between 2011 and 2019

**Year**	**Male age 50 - 59**		**Male age 60 - 69**		**Male age 70 - 79**		**Male age ≥ 80**	
	**Cases**	**Rates[1]**	**Cases**	**Rates[1]**	**Cases**	**Rates[1]**	**Cases**	**Rates[1]**
2011	6	0.36 (0.13 - 0.78)	12	1.36 (0.70 - 2.37)	18	3.36 (1.99 - 5.31)	40	12.93 (9.24 - 17.61)
2012	5	0.29 (0.10 - 0.69)	17	1.79 (1.04 - 2.87)	14	2.59 (1.41 - 4.34)	47	14.73 (10.82 - 19.59)
2013	8	0.46 (0.20 - 0.91)	19	1.87 (1.13 - 2.93)	21	3.82 (2.36 - 5.84)	31	9.53 (6.47 - 13.52)
2014	15	0.85 (0.48 - 1.41)	17	1.57 (0.92 - 2.52)	29	5.19 (3.47 - 7.45)	50	15.19 (11.28 - 20.03)
2015	15	0.85 (0.47 - 1.40)	27	2.33 (1.53 - 3.39)	46	8.19 (5.99 - 10.92)	76	22.89 (18.04 - 28.65)
2016	39	2.19 (1.56 - 2.99)	124	9.96 (8.29 - 11.88)	145	25.78 (21.75 - 30.33)	195	58.39 (50.48 - 67.18)
2017	53	2.97 (2.22 - 3.88)	136	10.33 (8.67 - 12.22)	192	33.52 (28.94 - 38.61)	264	78.56 (69.37 - 88.62)
2018	39	2.18 (1.55 - 2.98)	170	12.35 (10.56 - 14.35)	212	35.64 (31.00 - 40.77)	292	85.99 (76.41 - 96.44)
2019	43	2.40 (1.74 - 3.24)	166	11.60 (9.90 - 13.51)	247	39.45 (34.68 - 44.68)	283	82.15 (72.86 - 92.30)
**Mean**	25	1.41 (1.23 - 1.61)	76	6.58 (6.10 - 7.09)	103	18.10 (16.95 - 19.31)	142	43.04 (40.72 - 45.47)
**Year**	**Female age 50 - 59**		**Female age 60 - 69**		**Female age 70 - 79**		**Female age ≥ 80**	
	**Cases**	**Rates[1]**	**Cases**	**Rates[1]**	**Cases**	**Rates[1]**	**Cases**	**Rates[1]**
2011	10	0.58 (0.28 - 1.08)	14	1.48 (0.81 - 2.48)	6	0.96 (0.35 - 2.10)	8	2.58 (1.11 - 5.08)
2012	5	0.29 (0.09 - 0.67)	7	0.69 (0.28 - 1.42)	13	2.03 (1.08 - 3.48)	15	4.57 (2.56 - 7.54)
2013	8	0.45 (0.19 - 0.89)	13	1.20 (0.64 - 2.05)	12	1.83 (0.94 - 3.19)	18	5.20 (3.08 - 8.22)
2014	9	0.50 (0.23 - 0.95)	15	1.29 (0.72 - 2.13)	20	2.98 (1.82 - 4.61)	23	6.32 (3.74 - 8.90)
2015	13	0.71 (0.38 - 1.22)	16	1.28 (0.73 - 2.09)	20	2.96 (1.81 - 4.58)	24	6.26 (4.01 - 9.32)
2016	20	1.09 (0.67 - 1.68)	38	2.84 (2.01 - 3.89)	65	9.60 (7.41 - 12.24)	77	19.19 15.14 - 23.98)
2017	17	0.92 (0.54 - 1.48)	50	3.52 (2.61 - 4.64)	70	10.17 (7.93 - 12.85)	101	24.08 (19.61 - 29.26)
2018	26	1.41 (0.92 - 2.06)	65	4.36 (3.37 - 5.56)	81	11.37 (9.03 - 14.14)	82	18.68 (14.86 - 23.19)
2019	29	1.57 (1.04 - 2.25)	67	4.32 (3.47 - 5.48)	104	13.94 (11.39 - 16.89)	85	18.50 (14.78 - 22.88)
**Mean**	15	0.84 (0.71 - 1.00)	32	2.53 (2.25 - 2.84)	43	6.42 (5.80 - 7.09)	48	12.55 (11.39 - 13.79)

[1] Values indicate crude incidence rates (and the corresponding 95% confidence intervals in parentheses) per 100,000 persons of Taiwan's population on July 1 of each study year.

Supplemental Table 5 Annual **incidence cases** and **standardized incidence rates** of idiopathic pulmonary fibrosis in Taiwan between 2011 and 2019

Year	Overall		Male		Female		Male to female ratios
	Cases	Rates[1]	Cases	Rates[1]	Cases	Rates[1]	
2011	114	1.66 (1.36 – 1.97)	76	2.19 (1.70 – 2.69)	38	1.10 (0.75 – 1.45)	1.99 (1.35 – 2.94)
2012	123	1.72 (1.41 – 2.02)	83	2.34 (1.83 – 2.84)	40	1.09 (0.75 – 1.43)	2.14 (1.47 – 3.13)
2013	130	1.75 (1.45 – 2.05)	79	2.17 (1.69 – 2.65)	51	1.34 (0.97 – 1.71)	1.62 (1.14 – 2.30)
2014	178	2.30 (1.96 – 2.64)	111	2.95 (2.40 – 3.50)	67	1.68 (1.28 – 2.09)	1.75 (1.29 – 2.37)
2015	237	2.98 (2.60 – 3.36)	164	4.29 (3.63 – 4.94)	73	1.77 (1.36 – 2.17)	2.42 (1.84 – 3.19)
2016	703	8.59 (7.96 – 9.23)	503	12.88 (11.76 – 14.01)	200	4.69 (4.04 – 5.34)	2.75 (2.33 – 3.24)
2017	883	10.50 (9.81 – 11.19)	645	16.24 (14.99 – 17.50)	238	5.37 (4.68 – 6.05)	3.03 (2.61 – 3.51)
2018	967	11.09 (10.39 – 11.79)	713	17.45 (16.16 – 18.73)	254	5.53 (4.85 – 6.21)	3.15 (2.73 – 3.64)
2019	1024	11.35 (10.65 – 12.04)	739	17.55 (16.28 – 18.82)	285	5.99 (5.29 – 6.69)	2.93 (2.55 – 3.36)
**Mean**	484	6.09 (5.91 – 6.27)	345	9.04 (8.72 – 9.36)	138	3.35 (3.17 – 3.54)	2.70 (2.53 – 2.88)
**Year**	**Age group 50 - 59**		**Age group 60 - 69**		**Age group 70 - 79**		**Age group ≥ 80**	
	Cases	Rates[1]	Cases	Rates[1]	Cases	Rates[1]	Cases	Rates[1]
2011	16	0.47 (0.24 - 0.71)	26	1.42 (0.87 - 1.96)	24	2.07 (1.24 - 2.90)	48	7.74 (5.55 - 9.93)
2012	10	0.29 (0.11 - 0.47)	24	1.22 (0.73 - 1.71)	27	2.29 (1.42 - 3.15)	62	9.58 (7.19 - 11.96)
2013	16	0.46 (0.23 - 0.68)	32	1.52 (1.00 - 2.05)	33	2.74 (1.80 - 3.67)	49	7.30 (5.26 - 9.34)
2014	24	0.67 (0.40 - 0.94)	32	1.43 (0.93 - 1.92)	49	3.99 (2.87 - 5.10)	73	10.53 (8.12 - 12.95)
2015	28	0.78 (0.49 - 1.07)	43	1.79 (1.25 - 2.32)	66	5.34 (4.05 - 6.62)	100	13.98 (11.24 - 16.72)
2016	59	1.63 (1.21 - 2.05)	162	6.27 (5.30 - 7.23)	210	16.94 (14.65 - 19.23)	272	36.99 (32.60 - 41.39)
2017	70	1.93 (1.48 - 2.38)	186	6.79 (5.82 - 7.77)	262	20.77 (18.26 - 23.29)	365	48.31 (43.35 - 53.26)
2018	65	1.79 (1.35 - 2.22)	235	8.20 (7.15 - 9.25)	293	22.42 (19.85 - 24.99)	374	48.04 (43.18 - 52.91)
2019	72	1.98 (1.52 - 2.43)	233	7.81 (6.81 - 8.81)	351	25.58 (22.90 - 28.25)	368	45.78 (41.10 - 50.46)
**Mean**	40	1.12 (1.01 - 1.24)	108	4.48 (4.20 - 4.76)	146	11.75 (11.12 - 12.39)	190	26.65 (25.39 - 27.91)

[1] Values indicate the sex- and age-adjusted standardized incidence rates (and the corresponding 95% confidence intervals in parentheses) per 100,000 persons of Taiwan’s population on July 1, 2015 (the “standard population”).

Supplemental Table 6 Annual **incidence cases** and **standardized incidence rates** of idiopathic pulmonary fibrosis of **sex-specific age subgroups** in Taiwan between 2011 and 2019

Year	Male age 50 - 59		Male age 60 - 69		Male age 70 - 79		Male age ≥ 80	
	Cases	Rates[1]	Cases	Rates[1]	Cases	Rates[1]	Cases	Rates[1]
2011	6	0.36 (0.07 - 0.65)	12	1.36 (0.59 - 2.12)	18	3.36 (1.81 - 4.91)	40	12.93 (8.92 - 16.94)
2012	5	0.29 (0.04 - 0.55)	17	1.79 (0.94 - 2.64)	14	2.59 (1.23 - 3.94)	47	14.73 (10.52 - 18.94)
2013	8	0.46 (0.14 - 0.78)	19	1.87 (1.03 - 2.72)	21	3.82 (2.19 - 5.45)	31	9.53 (6.17 - 12.88)
2014	15	0.85 (0.42 - 1.29)	17	1.57 (0.83 - 2.32)	29	5.19 (3.30 - 7.07)	50	15.19 (10.98 - 19.41)
2015	15	0.85 (0.42 - 1.27)	27	2.33 (1.45 - 3.21)	46	8.19 (5.82 - 10.55)	76	22.89 (17.75 - 28.04)
2016	39	2.19 (1.50 - 2.88)	124	9.96 (8.21 - 11.72)	145	25.78 (21.58 - 29.97)	195	58.39 (50.19 - 66.58)
2017	53	2.97 (2.17 - 3.76)	136	10.33 (8.59 - 12.06)	192	33.52 (28.78 - 38.26)	264	78.56 (69.08 - 88.03)
2018	39	2.18 (1.50 - 2.86)	170	12.35 (10.49 - 14.21)	212	35.64 (30.84 - 40.44)	292	85.99 (76.13 - 95.85)
2019	43	2.40 (1.68 - 3.12)	166	11.60 (9.84 - 13.36)	247	39.45 (34.53 - 44.36)	283	82.15 (72.58 - 91.72)
**Mean**	25	1.41 (1.23 - 1.60)	76	6.58 (6.09 - 7.07)	103	18.10 (16.94 - 19.27)	142	43.04 (40.69 - 45.40)
**Year**	**Female age 50 - 59**		**Female age 60 - 69**		**Female age 70 - 79**		**Female age ≥ 80**	
	**Cases**	**Rates[1]**	**Cases**	**Rates[1]**	**Cases**	**Rates[1]**	**Cases**	**Rates[1]**
2011	10	0.58 (0.22 - 0.95)	14	1.48 (0.70 - 2.25)	6	0.96 (0.19 - 1.74)	8	2.58 (0.79 - 4.36)
2012	5	0.29 (0.04 - 0.54)	7	0.69 (0.18 - 1.20)	13	2.03 (0.93 - 3.14)	15	4.57 (2.26 - 6.88)
2013	8	0.45 (0.14 - 0.76)	13	1.20 (0.55 - 1.85)	12	1.83 (0.79 - 2.86)	18	5.20 (2.80 - 7.61)
2014	9	0.50 (0.17 - 0.82)	15	1.29 (0.64 - 1.95)	20	2.98 (1.68 - 4.29)	23	6.32 (3.74 - 8.90)
2015	13	0.71 (0.33 - 1.10)	16	1.28 (0.66 - 1.91)	20	2.96 (1.66 - 4.26)	24	6.26 (3.76 - 8.77)
2016	20	1.09 (0.61 - 1.57)	38	2.84 (1.93 - 3.74)	65	9.60 (7.27 - 11.94)	77	19.19 (14.90 - 23.47)
2017	17	0.92 (0.48 - 1.36)	50	3.52 (2.54 - 4.49)	70	10.17 (7.79 - 12.55)	101	24.08 (19.38 - 28.77)
2018	26	1.41 (0.87 - 1.95)	65	4.36 (3.30 - 5.42)	81	11.37 (8.90 - 13.85)	82	18.68 (14.64 - 22.73)
2019	29	1.57 (1.00 - 2.13)	67	4.32 (3.28 - 5.35)	104	13.94 (11.26 - 16.61)	85	18.50 (14.57 - 22.44)
**Mean**	15	0.84 (0.70 - 0.98)	32	2.53 (2.24 - 2.83)	43	6.42 (5.79 - 7.06)	48	12.55 (11.37 - 13.73)

[1] Values indicate the sex- and age-adjusted standardized incidence rates (and the corresponding 95% confidence intervals in parentheses) per 100,000 persons of Taiwan’s population on July 1, 2015 (the “standard population”).

Supplemental Table 7 Summary of results from the **Mann-Kendall test and Theil-Sen estimator** on longitudinal epidemiological trends of patients with idiopathic pulmonary fibrosis in Taiwan between 2011 and 2019

Trend-analysis of annual standardized **incidence** rates between 2011 and 2019				Trend-analysis of annual standardized **prevalence** rates between 2011 and 2019			
Trend to be analyzed	Tau value	Sen's slope	*p*-value	Trend to be analyzed	Tau value	Sen's slope	*p*-value
Total	1.000	1.381	< 0.001	Total	1.000	3.122	< 0.001
Male	0.889	2.233	0.001	Male	1.000	4.582	< 0.001
Female	0.944	0.690	< 0.001	Female	1.000	1.883	< 0.001
Age 50-59, total	0.833	0.237	0.002	Age 50-59, total	1.000	0.859	< 0.001
Age 50-59, male	0.761	0.313	0.006	Age 50-59, male	1.000	1.057	< 0.001
Age 50-59, female	0.778	0.160	0.005	Age 50-59, female	1.000	0.650	< 0.001
Age 60-69, total	0.833	0.967	0.002	Age 60-69, total	1.000	2.376	< 0.001
Age 60-69, male	0.833	1.533	0.002	Age 60-69, male	0.944	3.458	< 0.001
Age 60-69, female	0.667	0.519	0.016	Age 60-69, female	1.000	1.407	< 0.001
Age 70-79, total	1.000	3.138	< 0.001	Age 70-79, total	1.000	6.566	< 0.001
Age 70-79, male	0.944	4.978	< 0.001	Age 70-79, male	1.000	9.682	< 0.001
Age 70-79, female	0.889	1.625	0.001	Age 70-79, female	1.000	3.990	< 0.001
Age ≥ 80, total	0.722	5.804	0.009	Age ≥ 80, total	1.000	11.114	< 0.001
Age ≥ 80, male	0.833	10.676	0.002	Age ≥ 80, male	1.000	18.619	< 0.001
Age ≥ 80, female	0.667	2.258	0.016	Age ≥ 80, female	1.000	5.484	< 0.001

Trend-analysis of annual standardized **all-cause mortality** rates between 2011 and 2019				Trend-analysis of annual standardized **IPF-specific all-cause mortality** rates between 2011 and 2019			
Trend to be analyzed	Tau value	Sen's slope	*p*-value	Trend to be analyzed	Tau value	Sen's slope	*p*-value
Total	0.778	0.669	0.005	Total	−0.389	−0.867	0.175
Male	0.833	1.137	0.002	Male	−0.667	−1.188	0.016
Female	0.722	0.298	0.009	Female	−0.389	−1.189	0.175
Age 50-**69**, total[1]	0.889	0.161	0.001	Age 50-**69**, total[1]	−0.333	−0.967	0.251
Age 50-**69**, male[1]	0.944	0.241	< 0.001	Age 50-**69**, male[1]	−0.500	−1.147	0.076
Age 50-**69**, female[1]	0.556	0.046	0.048	Age 50-**69**, female[1]	−0.333	−0.865	0.251
Age 70-79, total	0.833	1.295	0.002	Age 70-79, total	−0.611	−0.583	0.029
Age 70-79, male	0.833	2.019	0.002	Age 70-79, male	−0.667	−1.506	0.016
Age 70-79, female	0.667	0.493	0.016	Age 70-79, female	−0.278	−0.219	0.348
Age ≥ 80, total	0.778	4.464	0.005	Age ≥ 80, total	−0.333	−1.063	0.251
Age ≥ 80, male	0.889	7.331	0.001	Age ≥ 80, male	−0.444	−1.884	0.118
Age ≥ 80, female	0.833	1.661	0.002	Age ≥ 80, female	−0.111	−0.339	0.754

1. Due to very small numbers of mortal cases, the two age-subgroups "50 - 59" and "60 - 69" were combined for analyses related to overall all-cause mortality rates and IPF-specific all-cause mortality rates.

Supplemental Table 8 Annual **prevalence cases** and **crude prevalence rates** of idiopathic pulmonary fibrosis in Taiwan between 2011 and 2019

Year	Overall		Male		Female		Male to female ratios
	Cases	Rates[1]	Cases	Rates[1]	Cases	Rates[1]	
2011	137	1.96 (1.65 - 2.32)	84	2.47 (1.97 - 3.06)	53	1.48 (1.11 - 1.93)	1.67 (1.19 – 2.36)
2012	204	2.82 (2.45 - 3.23)	116	3.31 (2.73 - 3.96)	88	2.36 (1.89 - 2.91)	1.40 (1.06 – 1.85)
2013	279	3.73 (3.30 - 4.19)	160	4.42 (3.76 - 5.16)	119	3.08 (2.55 - 3.69)	1.44 (1.13 – 1.82)
2014	383	4.96 (4.47 - 5.48)	219	5.88 (5.13 - 6.71)	164	4.10 (3.50 - 4.78)	1.43 (1.17 – 1.75)
2015	481	6.05 (5.52 - 6.61)	275	7.19 (6.36 - 8.09)	206	4.99 (4.33 - 5.72)	1.44 (1.20 – 1.73)
2016	893	10.92 (10.22 - 11.66)	557	14.20 (13.04 - 15.43)	336	7.90 (7.08 - 8.79)	1.80 (1.57 – 2.06)
2017	1352	16.12 (15.27 - 17.00)	893	22.25 (20.82 - 23.77)	459	10.49 (9.56 - 11.50)	2.12 (1.89 – 2.37)
2018	1933	22.50 (21.51 - 23.53)	1301	31.73 (30.03 - 33.50)	632	14.08 (13.00 - 15.22)	2.25 (2.05 – 2.48)
2019	2457	27.92 (26.82 - 29.04)	1675	39.97 (38.08 - 41.93)	782	16.96 (15.79 - 18.19)	2.36 (2.16 – 2.56)
**Mean**	902	11.38 (11.13 - 11.63)	587	15.39 (14.98 - 15.81)	315	7.67 (7.38 - 7.95)	2.01 (1.91 – 2.11)

Year	Age group 50 - 59		Age group 60 - 69		Age group 70 - 79		Age group ≥ 80	
	Cases	Rates[1]	Cases	Rates[1]	Cases	Rates[1]	Cases	Rates[1]
2011	25	0.74 (0.48 - 1.09)	29	1.58 (1.06 - 2.27)	36	3.11 (2.18 - 4.31)	47	7.58 (5.57 - 10.08)
2012	37	1.08 (0.76 - 1.48)	42	2.14 (1.54 - 2.89)	56	4.74 (3.58 - 6.16)	69	10.66 (8.29 - 13.49)
2013	48	1.37 (1.01 - 1.81)	59	2.81 (2.14 - 3.63)	73	6.05 (4.74 - 7.61)	99	14.75 (11.99 - 17.96)
2014	69	1.94 (1.51 - 2.45)	87	3.88 (3.11 - 4.79)	97	7.89 (6.40 - 9.63)	130	18.75 (15.67 - 22.27)
2015	87	2.42 (1.94 - 2.98)	104	4.32 (3.53 - 5.24)	136	10.99 (9.22 - 13.00)	154	21.53 (18.27 - 25.22)
2016	132	3.65 (3.05 - 4.33)	216	8.36 (7.28 - 9.55)	260	20.98 (18.51 - 23.69)	285	38.76 (34.39 - 43.53)
2017	175	4.82 (4.13 - 5.59)	322	11.76 (10.51 - 13.12)	406	32.19 (29.13 - 35.48)	449	59.43 (54.06 - 65.18)
2018	238	6.54 (5.74 - 7.43)	495	17.27 (15.78 - 18.86)	581	44.45 (40.91 - 48.22)	619	79.52 (73.38 - 86.03)
2019	273	7.50 (6.63 - 8.44)	646	21.66 (20.02 - 23.39)	766	55.81 (51.93 - 59.91)	772	96.04 (89.38 - 103.05)
**Mean**	120	3.39 (3.19 - 3.59)	222	9.21 (8.81 - 9.62)	268	21.54 (20.69 - 22.42)	292	40.87 (39.32 - 42.47)

[1] Values indicate crude prevalence rates (and the corresponding 95% confidence intervals in parentheses) per 100,000 persons of Taiwan's population on July 1 of each study year.

Supplemental Table 9 Annual **prevalence cases** and **crude prevalence rates** of idiopathic pulmonary fibrosis of **sex-specific age subgroups** in Taiwan between 2011 and 2019

**Year**	**Male age 50 - 59**		**Male age 60 - 69**		**Male age 70 - 79**		**Male age ≥ 80**	
	**Cases**	**Rates[1]**	**Cases**	**Rates[1]**	**Cases**	**Rates[1]**	**Cases**	**Rates[1]**
2011	10	0.60 (0.29 - 1.10)	14	1.58 (0.86 - 2.65)	21	3.92 (2.43 - 5.99)	39	12.61 (8.96 - 17.23)
2012	13	0.76 (0.41 - 1.31)	20	2.11 (1.29 - 3.25)	32	5.91 (4.04 - 8.35)	51	15.98 (11.90 - 21.02)
2013	18	1.04 (0.62 - 1.64)	31	3.06 (2.08 - 4.34)	40	7.28 (5.20 - 9.91)	71	21.82 (17.04 - 27.52)
2014	26	1.48 (0.97 - 2.17)	51	4.72 (3.51 - 6.21)	54	9.66 (7.26 - 12.60)	88	26.74 (21.45 - 32.94)
2015	36	2.03 (1.42 - 2.81)	54	4.66 (3.50 - 6.08)	79	14.06 (11.13 - 17.52)	106	31.93 (26.14 - 38.61)
2016	68	3.82 (2.96 - 4.84)	137	11.01 (9.24 - 13.01)	155	27.55 (23.39 - 32.24)	197	58.98 (51.04 - 67.82)
2017	96	5.37 (4.35 - 6.56)	213	16.17 (14.07 - 18.50)	264	46.09 (40.69 - 51.99)	320	95.22 (85.08 - 106.24)
2018	140	7.82 (6.58 - 9.23)	336	24.41 (21.87 - 27.16)	380	63.88 (57.62 - 70.64)	445	131.05 (119.16 - 143.80)
2019	156	8.72 (7.40 - 10.20)	439	30.68 (27.88 - 33.69)	516	82.40 (75.45 - 89.83)	564	163.72 (150.50 - 177.79)
**Mean**	62	3.57 (3.28 - 3.88)	144	12.38 (11.72 - 13.08)	171	30.19 (28.70 - 31.74)	209	63.35 (60.52 - 66.28)
**Year**	**Female age 50 - 59**		**Female age 60 - 69**		**Female age 70 - 79**		**Female age ≥ 80**	
	**Cases**	**Rates[1]**	**Cases**	**Rates[1]**	**Cases**	**Rates[1]**	**Cases**	**Rates[1]**
2011	15	0.88 (0.49 - 1.45)	15	1.58 (0.89 - 2.61)	15	2.41 (1.35 - 3.98)	8	2.58 (1.11 - 5.08)
2012	24	1.38 (0.88 - 2.05)	22	2.16 (1.36 - 3.28)	24	3.75 (2.40 - 5.58)	18	5.48 (3.25 - 8.67)
2013	30	1.69 (1.14 - 2.41)	28	2.58 (1.71 - 3.73)	33	5.03 (3.46 - 7.06)	28	8.09 (5.38 - 11.70)
2014	43	2.38 (1.72 - 3.21)	36	3.11 (2.17 - 4.30)	43	6.42 (4.64 - 8.64)	42	11.54 (8.31 - 15.59)
2015	51	2.80 (2.08 - 3.68)	50	4.01 (2.98 - 5.29)	57	8.44 (6.39 - 10.94)	48	12.53 (9.24 - 16.61)
2016	64	3.48 (2.68 - 4.45)	79	5.90 (4.67 - 7.35)	105	15.51 (12.69 - 18.78)	88	21.93 (17.59 - 27.02)
2017	79	4.28 (3.39 - 5.34)	109	7.67 (6.30 - 9.25)	142	20.63 (17.37 - 24.31)	129	30.75 (25.68 - 36.54)
2018	98	5.30 (4.30 - 6.46)	159	10.67 (9.08 - 12.47)	201	28.23 (24.46 - 32.41)	174	39.65 (33.98 - 45.99)
2019	117	6.31 (5.22 - 7.57)	207	13.34 (11.58 - 15.29)	250	33.50 (29.48 - 37.92)	208	45.28 (39.34 - 51.87)
**Mean**	58	3.21 (2.94 - 3.50)	78	6.26 (5.81 - 6.74)	97	14.29 (13.36 - 15.27)	83	21.53 (20.01 - 23.14)

[1] Values indicate crude prevalence rates (and the corresponding 95% confidence intervals in parentheses) per 100,000 persons of Taiwan's population on July 1 of each study year.

Supplemental Table 10 Annual **prevalence cases** and **standardized prevalence rates** of idiopathic pulmonary fibrosis in Taiwan between 2011 and 2019

Year	Overall		Male		Female		Male to female ratios
	Cases	Rates[1]	Cases	Rates[1]	Cases	Rates[1]	
2011	137	1.98 (1.65 - 2.31)	84	2.43 (1.91 - 2.95)	53	1.50 (1.09 - 1.90)	1.62 (1.15 - 2.29)
2012	204	2.83 (2.44 - 3.22)	116	3.25 (2.66 - 3.84)	88	2.38 (1.89 - 2.88)	1.36 (1.03 - 1.80)
2013	279	3.74 (3.30 - 4.17)	160	4.37 (3.69 - 5.05)	119	3.10 (2.54 - 3.66)	1.41 (1.11 - 1.79)
2014	383	4.96 (4.47 - 5.46)	219	5.85 (5.08 - 6.63)	164	4.11 (3.48 - 4.74)	1.42 (1.16 - 1.74)
2015	481	6.16 (5.61 - 6.71)	275	7.19 (6.34 - 8.04)	206	5.16 (4.46 - 5.87)	1.39 (1.16 - 1.67)
2016	893	10.92 (10.21 - 11.64)	557	14.27 (13.08 - 15.45)	336	7.89 (7.05 - 8.74)	1.81 (1.58 - 2.07)
2017	1352	16.08 (15.23 - 16.94)	893	22.42 (20.95 - 23.89)	459	10.43 (9.48 - 11.39)	2.15 (1.92 - 2.41)
2018	1933	22.24 (21.25 - 23.23)	1301	31.77 (30.04 - 33.50)	632	13.86 (12.78 - 14.94)	2.29 (2.08 - 2.52)
2019	2457	27.25 (26.17 - 28.33)	1675	39.64 (37.74 - 41.54)	782	16.50 (15.34 - 17.66)	2.40 (2.21 - 2.62)
**Mean**	902	11.34 (11.17 - 11.52)	587	15.34 (14.92 - 15.75)	315	7.64 (7.36 - 7.92)	2.01 (1.92 - 2.10)

Year	Age group 50 - 59		Age group 60 - 69		Age group 70 - 79		Age group ≥ 80	
	Cases	Rates[1]	Cases	Rates[1]	Cases	Rates[1]	Cases	Rates[1]
2011	25	0.74 (0.45 - 1.03)	29	1.58 (1.01 - 2.16)	36	3.11 (2.09 - 4.13)	47	7.58 (5.41 - 9.75)
2012	37	1.08 (0.73 - 1.42)	42	2.14 (1.49 - 2.78)	56	4.74 (3.50 - 5.98)	69	10.66 (8.14 - 13.17)
2013	48	1.37 (0.98 - 1.76)	59	2.81 (2.09 - 3.53)	73	6.05 (4.66 - 7.44)	99	14.75 (11.84 - 17.65)
2014	69	1.94 (1.48 - 2.39)	87	3.88 (3.07 - 4.70)	97	7.89 (6.32 - 9.46)	130	18.75 (15.53 - 21.98)
2015	87	2.43 (1.92 - 2.94)	104	4.49 (3.62 - 5.35)	136	11.04 (9.18 - 12.89)	154	22.12 (18.63 - 25.62)
2016	132	3.65 (3.03 - 4.27)	216	8.36 (7.24 - 9.47)	260	20.98 (18.43 - 23.53)	285	38.76 (34.26 - 43.26)
2017	175	4.82 (4.11 - 5.53)	322	11.76 (10.48 - 13.04)	406	32.19 (29.06 - 35.32)	449	59.43 (53.93 - 64.92)
2018	238	6.54 (5.71 - 7.37)	495	17.27 (15.75 - 18.79)	581	44.45 (40.84 - 48.07)	619	79.52 (73.26 - 85.78)
2019	273	7.50 (6.61 - 8.38)	646	21.66 (19.99 - 23.33)	766	55.81 (51.86 - 59.77)	772	96.04 (89.27 - 102.81)
**Mean**	120	3.39 (3.18 - 3.59)	222	9.21 (8.81 - 9.61)	268	21.54 (20.68 - 22.40)	292	40.87 (39.31 - 42.44)

[1] Values indicate the sex- and age-adjusted standardized prevalence rates (and the corresponding 95% confidence intervals in parentheses) per 100,000 persons of Taiwan’s total population on July 1, 2015 (the “standard population”).

Supplemental Table 11 Annual **prevalence cases** and **standardized prevalence rates** of idiopathic pulmonary fibrosis of **sex-specific age subgroups** in Taiwan between 2011 and 2019

**Year**	**Male age 50 - 59**		**Male age 60 - 69**		**Male age 70 - 79**		**Male age ≥ 80**	
	**Cases**	**Rates[1]**	**Cases**	**Rates[1]**	**Cases**	**Rates[1]**	**Cases**	**Rates[1]**
2011	10	0.60 (0.23 - 0.97)	14	1.58 (0.75 - 2.41)	21	3.92 (2.24 - 5.60)	39	12.61 (8.65 - 16.56)
2012	13	0.76 (0.35 - 1.18)	20	2.11 (1.18 - 3.03)	32	5.91 (3.86 - 7.96)	51	15.98 (11.60 - 20.37)
2013	18	1.04 (0.56 - 1.52)	31	3.06 (1.98 - 4.14)	40	7.28 (5.02 - 9.53)	71	21.82 (16.75 - 26.90)
2014	26	1.48 (0.91 - 2.05)	51	4.72 (3.43 - 6.02)	54	9.66 (7.08 - 12.23)	88	26.74 (21.15 - 32.33)
2015	36	2.03 (1.37 - 2.69)	54	4.66 (3.42 - 5.90)	79	14.06 (10.96 - 17.16)	106	31.93 (25.85 - 38.01)
2016	68	3.82 (2.91 - 4.72)	137	11.01 (9.16 - 12.85)	155	27.55 (23.22 - 31.89)	197	58.98 (50.75 - 67.22)
2017	96	5.37 (4.30 - 6.45)	213	16.17 (14.00 - 18.35)	264	46.09 (40.53 - 51.64)	320	95.22 (84.79 - 105.65)
2018	140	7.82 (6.53 - 9.12)	336	24.41 (21.80 - 27.02)	380	63.88 (57.46 - 70.30)	445	131.05 (118.88 - 143.21)
2019	156	8.72 (7.35 - 10.09)	439	30.68 (27.81 - 33.55)	516	82.40 (75.30 - 89.51)	564	163.72 (150.22 - 177.22)
**Mean**	62	3.57 (3.27 - 3.86)	144	12.38 (11.71 - 13.06)	171	30.19 (28.68 - 31.70)	209	63.35 (60.49 - 66.22)
**Year**	**Female age 50 - 59**		**Female age 60 - 69**		**Female age 70 - 79**		**Female age ≥ 80**	
	**Cases**	**Rates[1]**	**Cases**	**Rates[1]**	**Cases**	**Rates[1]**	**Cases**	**Rates[1]**
2011	15	0.88 (0.43 - 1.32)	15	1.58 (0.78 - 2.39)	15	2.41 (1.19 - 3.63)	8	2.58 (0.79 - 4.36)
2012	24	1.38 (0.83 - 1.93)	22	2.16 (1.26 - 3.07)	24	3.75 (2.25 - 5.25)	18	5.48 (2.95 - 8.02)
2013	30	1.69 (1.08 - 2.29)	28	2.58 (1.62 - 3.53)	33	5.03 (3.31 - 6.74)	28	8.09 (5.10 - 11.09)
2014	43	2.38 (1.67 - 3.09)	36	3.11 (2.09 - 4.12)	43	6.42 (4.50 - 8.33)	42	11.54 (8.05 - 15.02)
2015	51	2.82 (2.05 - 3.60)	50	4.31 (3.12 - 5.51)	57	8.50 (6.30 - 10.71)	48	13.18 (9.45 - 16.91)
2016	64	3.48 (2.63 - 4.34)	79	5.90 (4.60 - 7.20)	105	15.51 (12.55 - 18.48)	88	21.93 (17.35 - 26.51)
2017	79	4.28 (3.34 - 5.23)	109	7.67 (6.23 - 9.11)	142	20.63 (17.23 - 24.02)	129	30.75 (25.45 - 36.06)
2018	98	5.30 (4.25 - 6.35)	159	10.67 (9.02 - 12.33)	201	28.23 (24.32 - 32.13)	174	39.65 (33.76 - 45.54)
2019	117	6.31 (5.17 - 7.46)	207	13.34 (11.52 - 15.16)	250	33.50 (29.35 - 37.65)	208	45.28 (39.13 - 51.43)
**Mean**	58	3.21 (2.93 - 3.48)	78	6.26 (5.80 - 6.73)	97	14.29 (13.34 - 15.24)	83	21.53 (19.98 - 23.08)

[1] Values indicate the sex- and age-adjusted standardized prevalence rates (and the corresponding 95% confidence intervals in parentheses) per 100,000 persons of Taiwan's total population on July 1, 2015 (the "standard population").

Supplemental Table 12 Annual **mortal cases** and **standardized all-cause mortality rates** of idiopathic pulmonary fibrosis of **sex-specific age subgroups** in Taiwan between 2011 and 2019

Year	Overall		Male		Female		Age group 50 - 69[1]		Age group 70 - 79		Age group ≥ 80	
	Cases	Rates[2]	Cases	Rates[2]	Cases	Rates[2]	Cases	Rates[2]	Cases	Rates[2]	Cases	Rates[2]
2011	55	0.76 (0.56–0.96)	42	1.18 (1.01–1.35)	13	0.37 (0.26–0.47)	8	0.15 (0.07–0.23)	16	1.37 (1.26–1.47)	31	4.79 (4.64 – 4.94)
2012	49	0.66 (0.47–0.84)	39	1.07 (0.91–1.23)	10	0.27 (0.18–0.36)	10	0.18 (0.10–0.27)	8	0.67 (0.60–0.75)	31	4.58 (4.44 – 4.73)
2013	45	0.59 (0.42–0.77)	31	0.84 (0.69–0.98)	14	0.37 (0.27–0.47)	9	0.16 (0.08–0.24)	13	1.08 (0.99–1.17)	23	3.38 (3.26 – 3.50)
2014	89	1.14 (0.90–1.38)	64	1.69 (1.50–1.89)	25	0.63 (0.50–0.76)	19	0.33 (0.22–0.44)	16	1.30 (1.20–1.40)	54	7.70 (7.51 – 7.86)
2015	88	1.11 (0.88–1.34)	73	1.91 (1.70–2.12)	15	0.36 (0.27–0.46)	12	0.20 (0.11–0.29)	21	1.70 (1.58–1.81)	55	7.69 (7.50 – 7.87)
2016	204	2.53 (2.18–2.87)	151	3.91 (3.61–4.21)	53	1.24 (1.07–1.42)	30	0.48 (0.35–0.61)	49	3.95 (3.78–4.13)	125	17.21 (16.94 – 17.48)
2017	307	3.73 (3.31–4.14)	232	5.93 (5.56–6.29)	75	1.69 (1.49–1.89)	54	0.85 (0.68–1.02)	84	6.66 (6.44–6.88)	169	22.80 (22.50 – 23.11)
2018	426	5.06 (4.58–5.55)	321	8.06 (7.64–8.49)	105	2.29 (2.06–2.51)	72	1.11 (0.92–1.30)	108	8.25 (8.01–8.50)	246	32.75 (32.39 – 33.12)
2019	492	5.69 (5.19–6.19)	378	9.24 (8.79–9.69)	114	2.40 (2.17–2.63)	92	1.39 (1.18–1.61)	140	10.18 (9.92–10.44)	260	34.01 (33.63 – 34.38)
**Mean**	195	2.36 (1.37–3.36)	148	3.76 (2.88–4.63)	47	1.07 (0.59–1.55)	34	0.54 (0.13–0.95)	51	3.91 (3.40–4.41)	110	14.99 (14.23–15.75)
**Year**	**Male age 50 - 69[1]**		**Male age 70 - 79**		**Male age ≥ 80**		**Female age 50 - 69[1]**		**Female age 70 - 79**		**Female age ≥ 80**	
	**Cases**	**Rates[2]**	**Cases**	**Rates[2]**	**Cases**	**Rates[2]**	**Cases**	**Rates[2]**	**Cases**	**Rates[2]**	**Cases**	**Rates[2]**
2011	5	0.20 (0.13–0.26)	12	2.24 (2.15–2.33)	25	8.08 (7.95–8.21)	3	0.11 (0.06–0.16)	4	0.64 (0.59–0.70)	6	1.93 (1.86–2.01)
2012	6	0.23 (0.16–0.29)	6	1.11 (1.05–1.17)	27	8.46 (8.33–8.60)	4	0.15 (0.09–0.20)	2	0.31 (0.28–0.35)	4	1.22 (1.16–1.28)
2013	7	0.26 (0.19–0.32)	9	1.64 (1.56–1.71)	15	4.61 (4.51–4.71)	2	0.07 (0.03–0.11)	4	0.61 (0.56–0.66)	8	2.31 (2.24–2.39)
2014	13	0.46 (0.37–0.55)	10	1.79 (1.71–1.87)	41	12.46 (12.30–12.62)	6	0.20 (0.14–0.26)	6	0.90 (0.83–0.96)	13	3.57 (3.48–3.66)
2015	11	0.38 (0.29–0.46)	17	3.03 (2.92–3.13)	45	13.55 (13.39–13.72)	1	0.03 (0.01–0.06)	4	0.59 (0.54–0.67)	10	2.61 (2.53–2.69)
2016	22	0.73 (0.61–0.84)	34	6.04 (5.90–6.19)	95	28.44 (28.21–28.68)	8	0.25 (0.18–0.32)	15	2.22 (2.12–2.31)	30	7.48 (7.35–7.60)
2017	46	1.48 (1.32–1.64)	69	12.05 (11.84–12.25)	117	34.82 (34.55–35.08)	8	0.24 (0.18–0.31)	15	2.18 (2.09–2.27)	52	12.40 (12.23–12.56)
2018	51	1.61 (1.45–1.77)	84	14.12 (13.91–14.33)	186	54.77 (54.45–55.10)	21	0.63 (0.53–0.73)	24	3.37 (3.26–3.48)	60	13.67 (13.50–13.84)
2019	75	2.33 (2.13–2.52)	100	15.97 (15.75–16.19)	203	58.93 (58.59–59.27)	17	0.50 (0.41–0.59)	40	5.36 (5.22–5.50)	57	12.41 (12.25–12.56)
**Mean**	26	0.85 (0.49–1.21)	38	6.44 (6.01–6.88)	84	24.90 (24.24–25.57)	8	0.24 (0.04–0.44)	13	1.80 (1.55–2.05)	27	6.40 (6.05–6.75)

[1] Due to the very small numbers of mortal cases, for the calculation of mortality rates, the age-subgroups “50 - 59” and “60 – 69” were combined.

[2] Values indicate the sex- and age-adjusted standardized mortality rates (and the corresponding 95% confidence intervals in parentheses) per 100,000 persons of Taiwan’s population on July 1, 2015 (the “standard population”).

Supplemental Table 13 Annual **standardized IPF-specific all-cause mortality rates** of patients with idiopathic pulmonary fibrosis in Taiwan between 2011 and 2019

Year	Overall	Male	Female	Age group 50 - 69[1]	Age group 70 - 79	Age group ≥ 80
2011	26.22 (26.19 – 26.25)	32.04 (31.99 – 32.08)	20.83 (20.79 – 20.87)	19.00 (18.96 – 19.03)	46.06 (45.97 – 46.15)	52.58 (52.46 – 52.69)
2012	16.01 (15.99 – 16.04)	19.06 (19.04 – 19.12)	13.17 (13.14 – 13.20)	14.74 (14.71 – 14.77)	15.86 (15.80 – 15.93)	26.92 (26.82 – 27.02)
2013	10.56 (10.54 – 10.58)	14.05 (14.02 – 14.09)	7.32 (7.29 – 7.34)	8.27 (8.25 – 8.29)	16.55 (16.48 – 16.62)	19.38 (19.29 – 19.47)
2014	14.33 (14.31 – 14.35)	18.00 (17.96 – 18.04)	10.93 (10.90 – 10.96)	12.90 (12.87 – 12.92)	13.96 (13.89 – 14.02)	27.00 (26.89 – 27.10)
2015	7.94 (7.92 – 7.96)	13.02 (12.99 – 13.05)	3.23 (3.21 – 3.24)	5.89 (5.87 – 5.91)	11.12 (11.06 – 11.17)	19.63 (19.54 – 19.72)
2016	9.72 (9.70 – 9.74)	11.43 (11.40 – 11.47)	8.14 (8.11 – 8.16)	7.20 (7.18 – 7.22)	13.60 (13.54 – 13.67)	24.17 (24.07 – 24.27)
2017	9.82 (9.80 – 9.84)	13.25 (13.22 – 13.29)	6.63 (6.61 – 6.66)	7.65 (7.62 – 7.67)	12.86 (12.80 – 12.92)	22.77 (22.68 – 22.87)
2018	10.65 (10.63 – 10.67)	11.81 (11.78 – 11.84)	9.58 (9.55 – 9.61)	8.67 (8.64 – 8.69)	12.78 (12.72 – 12.84)	23.66 (23.56 – 23.76)
2019	10.21 (10.19 – 10.23)	12.96 (12.92 – 12.99)	7.66 (7.64 – 7.69)	8.33 (8.31 – 8.35)	13.46 (13.40 – 13.52)	20.39 (20.29 – 20.48)
**Mean**	12.83 (12.82 – 12.84)	16.18 (16.17 – 16.19)	9.72 (9.71 – 9.73)	10.29 (10.28 – 10.30)	17.36 (17.34 – 17.38)	26.28 (26.24 – 26.31)
**Year**	**Male age 50 - 69[1]**	**Male age 70 - 79**	**Male age ≥ 80**	**Female age 50 - 69[1]**	**Female age 70 - 79**	**Female age ≥ 80**
2011	26.32 (26.27 – 26.37)	48.00 (47.87 – 48.13)	55.56 (55.39 – 55.72)	12.00 (11.96 – 12.04)	44.44 (44.33 – 44.56)	50.00 (49.84 – 50.16)
2012	16.22 (16.17 – 16.26)	24.00 (23.89 – 24.11)	36.00 (35.84 – 36.16)	13.33 (13.30 – 13.37)	9.09 (9.02 – 9.16)	19.05 (18.92 – 19.17)
2013	12.28 (12.24 – 12.32)	20.93 (20.82 – 21.04)	18.07 (17.94 – 18.20)	4.44 (4.42 – 4.47)	12.90 (12.82 – 12.98)	20.51 (20.39 – 20.64)
2014	16.89 (16.84 – 16.93)	15.38 (15.29 – 15.48)	32.28 (32.12 – 32.44)	9.09 (9.06 – 9.12)	12.77 (12.69 – 12.85)	22.41 (22.28 – 22.55)
2015	10.89 (10.86 – 10.93)	16.19 (16.09 – 16.29)	26.47 (26.32 – 26.62)	1.11 (1.10 – 1.12)	6.90 (6.84 – 6.96)	13.70 (13.59 – 13.81)
2016	8.80 (8.77 – 8.83)	15.18 (15.08 – 15.27)	28.36 (28.21 – 28.51)	5.67 (5.65 – 5.70)	12.30 (12.22 – 12.37)	20.55 (20.42 – 20.68)
2017	11.22 (11.18 – 11.26)	18.40 (18.30 – 18.50)	22.50 (22.36 – 22.64)	4.23 (4.21 – 4.26)	8.24 (8.18 – 8.31)	23.01 (22.88 – 23.14)
2018	9.41 (9.38 – 9.44)	16.12 (16.03 – 16.22)	27.29 (27.14 – 27.44)	7.95 (7.92 – 7.98)	10.00 (9.93 – 10.07)	21.90 (21.77 – 22.03)
2019	11.47 (11.43 – 11.50)	14.47 (14.38 – 14.56)	23.55 (23.41 – 23.69)	5.33 (5.30 – 5.35)	12.62 (12.54 – 12.70)	17.65 (17.53 – 17.77)
**Mean**	13.72 (13.71 – 13.73)	20.96 (20.93 – 21.00)	29.83 (29.78 – 29.88)	7.02 (7.01 – 7.03)	14.36 (14.33 – 14.39)	23.20 (23.15 – 23.24)

Values are in % (and the corresponding 95% confidence intervals in parentheses), indicating the sex- and age-adjusted standardized IPF-specific all-cause mortality rates.

[1] Due to the very small numbers of mortal cases, for the calculation of mortality rates, the age-subgroups “50 - 59” and “60 – 69” were combined.

IPF, idiopathic pulmonary fibrosis

Supplemental Table 14 The overall and seasonal distribution of causes of death of patients with Idiopathic pulmonary fibrosis in Taiwan between 2011 and 2019

Causes of death	Overall	Spring (Mar. – May)	Summer (Jun. – Aug.)	Fall (Sept. – Nov.)	Winter (Dec. – Feb.)
Infection	72	19	18	20	15
Respiratory (non-neoplastic)	917	220	236	233	228
Lung cancer	155	39	36	37	43
Other cancers	130	31	35	35	29
Hematologic	20	4	3	9	4
Cardiovascular	196	50	41	53	52
Neurological	78	26	17	16	19
Endocrine	40	14	5	10	11
Gastrointestinal	20	5	4	4	7
Hepatobiliary	25	7	6	10	2
Renal-genitourinary	45	13	10	10	12
Soft tissue and musculoskeletal	28	6	8	5	9
Suicide and trauma	29	8	8	7	6
**Total**	1755	442	427	449	437
**Specific etiologies of non-neoplastic respiratory causes of death**					
Progression of IPF				471	
Pneumonia				211	
Co-existing obstructive disorders				204	
Chronic respiratory failure				12	
Extrinsic agent-induced disorders				9	
Respiratory arrest				6	
Pleural disorders				4	

IPF, idiopathic pulmonary fibrosis.

Supplemental Table 15 Annual proportions of patients with idiopathic pulmonary fibrosis receiving at least one prescription of selected medications

Years	Azathioprine	Corticosteroids	Cyclophosphamide	Cyclosporine	Nintedanib	Pirfenidone
2011	0.45	37.50	0	0	0	0
2012	0.34	36.30	0.34	0	0	0
2013	0	31.64	0	0	0	0
2014	0.59	30.83	0.79	0.20	0	0
2015	0.46	28.59	0.46	0	0	0
2016	0.63	38.06	0.32	0	0	0
2017	0.36	34.96	0.51	0	12.01	0.46
2018	0.50	33.74	0.35	0.04	16.22	3.30
2019	0.75	33.06	0.50	0.03	16.66	5.61

Values (**in %**) were calculated using the following formula:
[(number of IPF patients receiving ≥ 1 prescription of an agent in a study year) / (total number of IPF patients alive in that same year)] x 100%

In accordance with the regulations set forth by Taiwan's Health and Welfare Data Science Center, Ministry of Health and Welfare, the distribution of case numbers among different groups relating to specific therapies or medications could not be presented if the case count in any group was less than 3.

IPF, idiopathic pulmonary fibrosis.

Supplemental Table 16 Summary of results from the **Mann-Kendall test and Theil-Sen estimator** on longitudinal trends of specific managements for patients with idiopathic pulmonary fibrosis in Taiwan between 2011 and 2019

**Trends to be analyzed**	**Tau value**	**Sen's slope**	***p*-value**
Annual rates of prescription claims for specific medication:			
Azathioprine	0.444	0.037	0.118
Cyclophosphamide	0.366	0.036	0.208
Cyclosporin	0.327	0.000	0.316
Nintedanib	0.764	2.200	0.012
Pirfenidone	0.764	0.135	0.012
Corticosteroids	−0.222	−0.500	0.466
Mean daily cortisone-equivalent dose per patient[1]	−1.000	−0.454	< 0.001
RF/IPF cases[2]	−0.722	−0.921	0.009
Annual rates of mechanical ventilation:			
IMV/total MV[3]	−0.944	−2.979	< 0.001
NIV/total MV[3]	0.944	2.979	< 0.001

[1.] Please refer to the main text for the formula standardizing the daily dosage per patient of various forms of corticosteroids to a cortisone-equivalent dosage.
[2.] Annual rates of respiratory failure, derived by dividing the number of IPF patients with respiratory failure in a study year by the total number of IPF patients alive in that same year.
[3.] The annual number of IPF patients receiving invasive, or non-invasive, mechanical ventilation in a study year over the total number of IPF patients receiving mechanical ventilation in that same year.
IMV, invasive mechanical ventilation; IPF, idiopathic pulmonary fibrosis; MV, mechanical ventilation; NIV, non-invasive mechanical ventilation; RF, respiratory failure.

Supplemental Table 17 Annual proportions of respiratory failure and invasive and noninvasive mechanical ventilation

Year	Respiratory failure		Invasive mechanical ventilation (IMV)		Non-invasive mechanical ventilation (NIV)	
	Cases	Rates (%)[1]	Cases of IMV	Proportions of (IMV + NIV) (%)[2]	Cases of NIV	Proportions of (IMV + NIV) (%)[3]
2011	29	21.5	23	65.7	12	34.3
2012	37	17.6	25	64.1	14	35.9
2013	40	13.4	35	62.5	21	37.5
2014	45	10.2	35	63.6	20	36.4
2015	69	11.6	41	60.3	27	39.7
2016	150	12.3	89	57.1	67	42.9
2017	219	11.5	111	47.0	125	53.0
2018	278	10.8	158	45.4	190	54.6
2019	307	9.7	159	44.2	201	55.8
**Mean**	130	13.2	75	56.7	75	43.3

[1] Derived by dividing the number of IPF patients developing respiratory failure in a study year by the total number of IPF patients alive in that same year.
[2] Derived by dividing the number of IPF patients receiving IMV in a study year by the total number of all IPF patients receiving mechanical ventilation in that same year.
[3] Derived by dividing the number of IPF patients receiving NIV in a study year by the total number of all IPF patients receiving mechanical ventilation in that same year.
IMV, invasive mechanical ventilation; IPF, idiopathic pulmonary fibrosis; NIV, non-invasive mechanical ventilation.

Supplemental Figure 1 Annual numbers of electronic news articles relating to "pulmonary fibrosis" from Taiwan's top 3 news agencies between 2000 and 2020

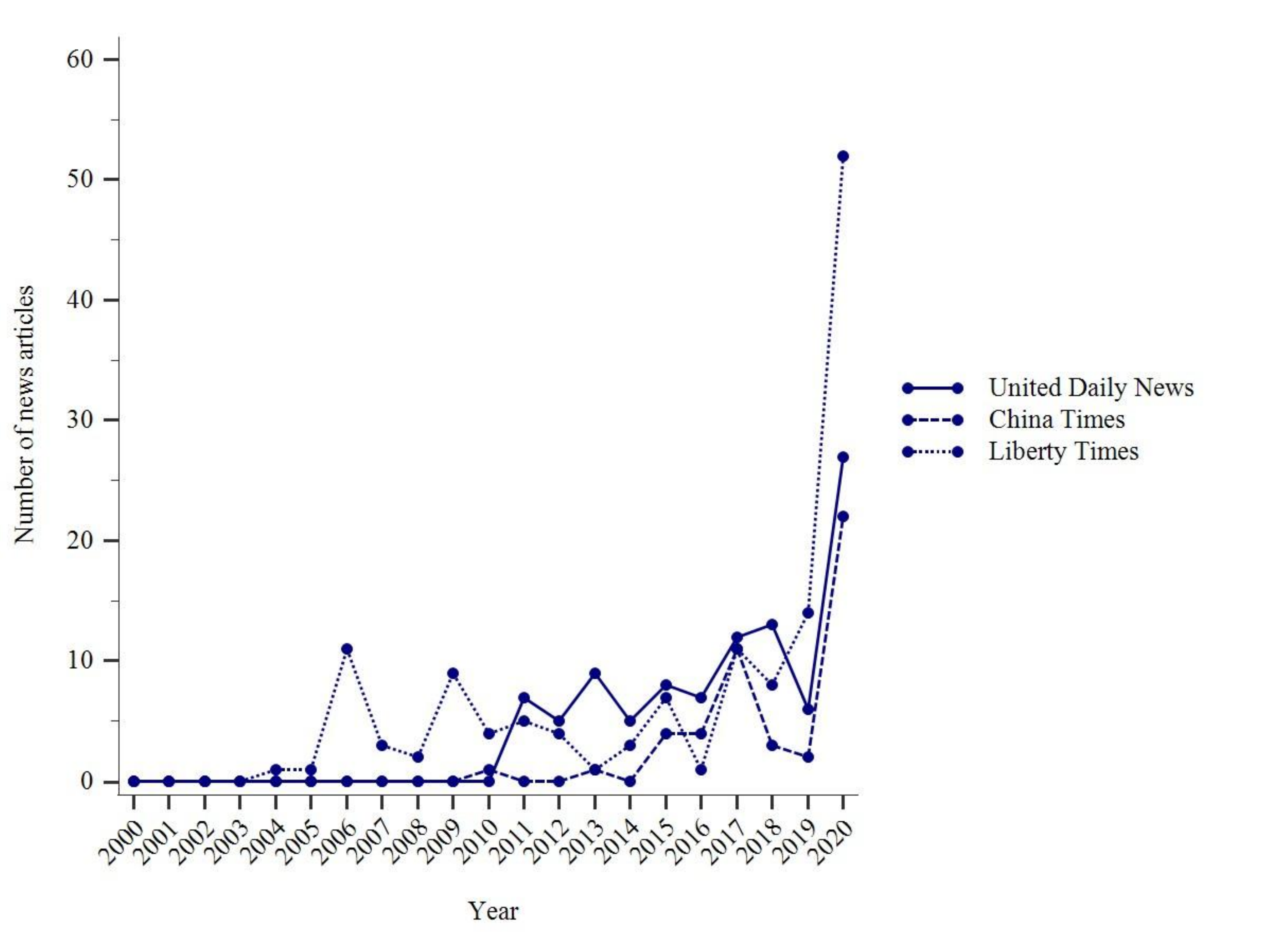

Trend analysis by Mann-Kendall test and Theil-Sen estimator			
**Newspaper Names**	**Tau value**	**Sen's slope**	***p*-value**
United Daily News (overall)	0.659	0.724	< 0.001
China Times (overall)	0.722	0.286	< 0.001
Liberty Times (overall)	0.752	0.857	< 0.001

Appendix 1: Validation process of the working definition of idiopathic pulmonary fibrosis (IPF) for this study

- Members of the multi-disciplinary team conducting the validation process (all from National Cheng Kung University Hospital, NCKUH):
  (1) Dr. Shen-Huan Wei and Dr. Chin-Wei Kuo (Division of Chest Medicine, Department of Internal Medicine)
  (2) Dr. Li-Ting Huang (Department of Diagnostic Radiology)
  (3) Dr. Chia-Tse Weng (Division of Allergy, Immunology and Rheumatology, Department of Internal Medicine)
- Source of patient clinical data: the electronic medical records of NCKUH – access to these medical records was part of the study protocol that has been approved by the Institutional Review Board of NCKUH (approval codes A-ER-109-321 and B-EX-111-034) and also by the Information Management Office of NCKUH.
- Working definitions of IPF to be validated – ***all*** 4 components must be satisfied to be classified as a case of IPF:
  (1) ≥ 1 inpatient or ambulatory/emergent claim with compatible ICD-9-CM codes (516.3 or 516.31) or ICD-10-CM codes (J84.112; starting from January 1, 2016);
  (2) age ≥ 50 years when the diagnostic code claim was first made;
  (3) ≥ 1 claim for a crucial diagnostic procedure (as listed in Supplemental Table 1) ***within*** one year ***before*** the initial diagnostic code claim;
  (4) no competing diagnostic code (as listed in Supplemental Table 2) was claimed ***within*** one year ***after*** the initial diagnostic code claim.
- Adjudication of true IPF or non-IPF - For each patient identified by the working definition, the complete electronic medical records (outpatient, inpatient, emergent visit) were carefully reviewed, with relevant data systematically recorded, for the integrated assessment to ascertain the pulmonary diagnosis. Attention was particularly paid to the following:
  (1) documented list of diagnoses (pulmonary and extrapulmonary; especially documented diagnoses of connective tissue disease, cancer, arrhythmia…etc.);
  (2) presence or absence of "Identity of Catastrophic Illness" as registered in the National Health Insurance ("Catastrophic Illness" included major connective tissue diseases, cancers, chronic respiratory failure with prolonged dependence on mechanical ventilation…etc.);
  (3) records/description of pulmonary and extra-pulmonary symptoms and physical signs;
  (4) medication lists (especially cancer-related therapies, immunosuppressants, biologics, anti-arrhythmic agents, antifibrotics…etc.);
  (5) documentation of cigarette smoking or special occupational / recreational / environmental exposures;
  (6) blood laboratory tests results (especially autoimmune serological tests);
  (7) pulmonary function measurements;

(8) radiographic images and formal written reports, especially those of chest CT scan;
(9) pathology report of lung biopsy or relevant extrapulmonary tissue biopsies (ex. salivary gland biopsy as for Sjogren syndrome; bone marrow biopsy as for lymphoma…etc.).

- We applied Diagnosis of Idiopathic Pulmonary Fibrosis An Official ATS/ERS/JRS/ALAT Clinical Practice Guideline (*Am J Respir Crit Care Med*. 2018;198(5):e44-e68) and the 2022 updates (*Am J Respir Crit Care Med*. 2022;205(9):e18-e47) to determine true cases of IPF.
- For the classification of non-IPF idiopathic or secondary ILDs, we applied the official American Thoracic Society/European Respiratory Society statement: Update of the international multidisciplinary classification of the idiopathic interstitial pneumonias (*Am J Respir Crit Care Med*. 2013;188(6):733-478).
- Disagreement among member specialists was solved by consensus.

- Summary flowchart of the validation process:

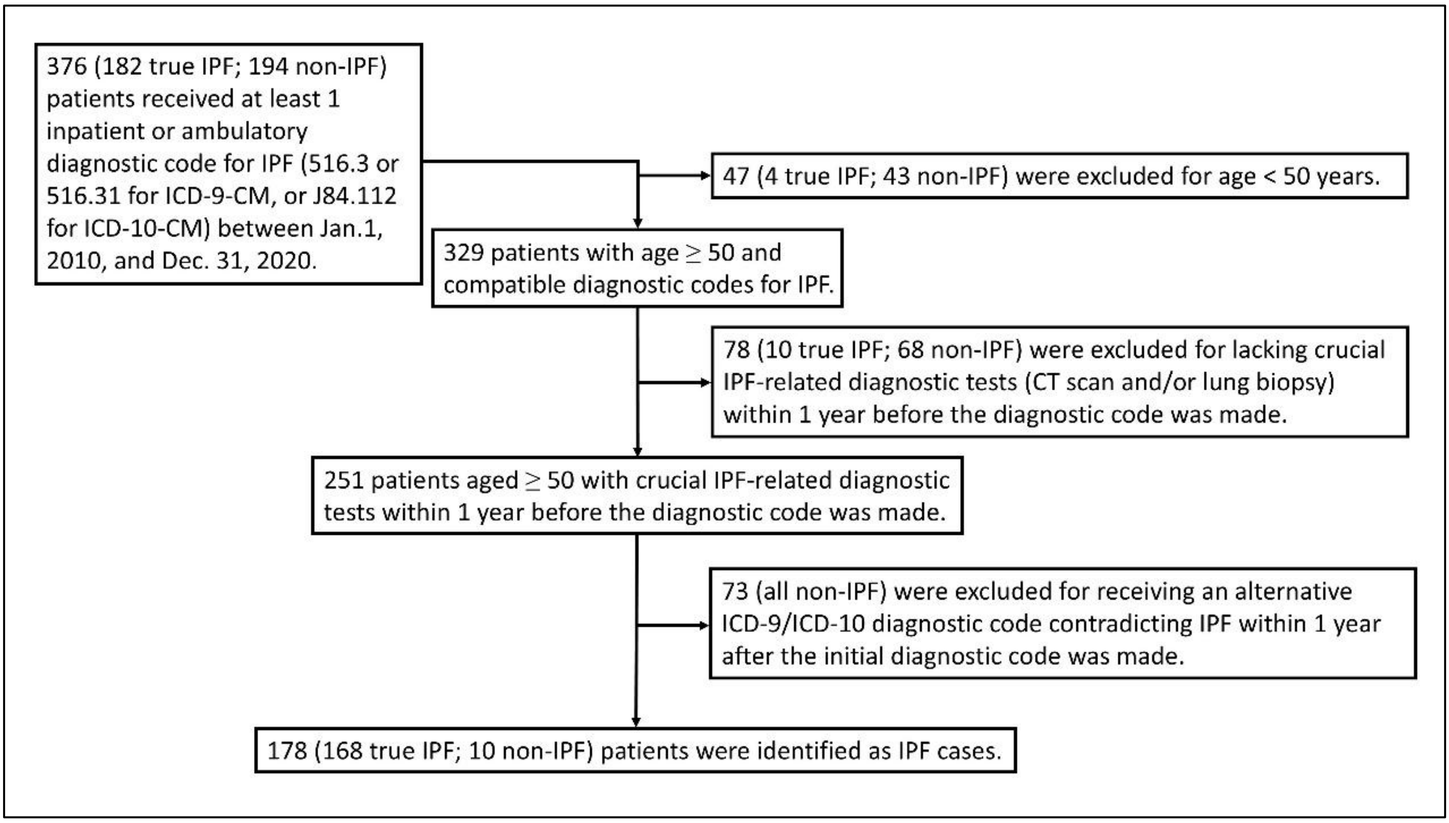

Note: Of the 78 patients excluded for not having crucial IPF-related diagnostic tests ***within one year*** prior to receiving the diagnostic code, 16 patients underwent chest CT scan and 2 underwent lung biopsy beyond this time interval.

Abbreviations: CT, computed tomography; ICD-9/-10-CM, International Classification of Disease, Nineth or Tenth Revision, Clinical Modification; IPF, idiopathic pulmonary fibrosis.

- Formula for the calculation of sensitivity, specificity, positive predictive value (PPV), and negative predictive value (NPV) of the working definition:

  Sensitivity = TP/(TP + FN) = 168 / [168+(4+10)] = 0.92 (92%)

  Specificity = TN/(TN + FP) = (43+68+73) / [(43+68+73]+10] = 0.95 (95%)

  PPV = TP/(TP + FP) = 168 / (168 + 10) = 0.94 (94%)

  NPV = TN/(TN + FN) = (43+68+73)/[(43+68+73) +(4+10)] = 0.93 (93%)

  TP: true positive – a true IPF case and also correctly identified as IPF by the working definition

  FP: false positive – a non-IPF case but falsely identified as IPF by the working definition

  TN: true negative – a non-IPF case and also correctly identified as non-IPF by the working definition

  FN: false negative – a true IPF case but falsely identified as non-IPF by the working definition

- Diagnoses of the cases that were considered as "non-IPF":

**Diagnoses**	**N. of patients**
ILDs secondary to connective tissue diseases	39
Other post-inflammatory fibrosis or focal scarring fibrosis	27
Infectious pneumonia	15
Bronchitis/bronchiolitis	13
Sequelae of prior pulmonary tuberculosis	11
Bronchiectasis	10
Atelectasis	9
Chronic obstructive pulmonary disease (COPD)	9
Idiopathic nonspecific interstitial pneumonia (iNSIP)	9
Organizing pneumonia (OP), cryptogenic or secondary	9
Emphysema	7
Lung-origin or metastatic cancer	7
Lung nodules	7
Pulmonary congestion / edema	7
Chronic hypersensitivity pneumonia	4
Asthma	3
Pneumoconiosis	1
Post-radiation fibrosis	1
Pulmonary alveolar proteinosis (PAP)	1
Total anomalous pulmonary venous return (TAPVR) post-surgery	1
No pulmonary pathology/disease	4
Total	194
